# Optimising strategies to address mental ill-health in doctors and medical students: ‘Care Under Pressure’ realist review and implementation guidance

**DOI:** 10.1186/s12916-020-01532-x

**Published:** 2020-04-08

**Authors:** Daniele Carrieri, Karen Mattick, Mark Pearson, Chrysanthi Papoutsi, Simon Briscoe, Geoff Wong, Mark Jackson

**Affiliations:** 1 College of Medicine and Health, University of Exeter, Exeter, UK; 2 Wellcome Centre for Cultures and Environments of Health, University of Exeter, Exeter, UK; 3 Wolfson Palliative Care Research Centre, Hull York Medical School, Faculty of Health Sciences, University of Hull, Hull, UK; 4 Nuffield Department of Primary Care Health Sciences, University of Oxford, Oxford, UK; 5 Exeter HS&DR Evidence Synthesis Centre, Institute of Health Research, College of Medicine and Health, University of Exeter, Exeter, UK

**Keywords:** Wellbeing, Doctors, Physicians, Medical students, Mental ill-health, Burnout, Distress, Intervention, Stress management, Coping, Prevention, Organisational culture, Job satisfaction

## Abstract

**Background:**

Mental ill-health in health professionals, including doctors, is a global and growing concern. The existing literature on interventions that offer support, advice and/or treatment to sick doctors has not yet been synthesised in a way that considers the complexity and heterogeneity of the interventions, and the many dimensions of the problem. We (1) reviewed interventions to tackle doctors’ and medical students’ mental ill-health and its impacts on the clinical workforce and patient care—drawing on diverse literature sources and engaging iteratively with diverse stakeholder perspectives—and (2) produced recommendations that support the tailoring, implementation, monitoring and evaluation of contextually sensitive strategies to tackle mental ill-health and its impacts.

**Methods:**

Realist literature review consistent with the RAMESES quality and reporting standards. Sources for inclusion were identified through bibliographic database searches supplemented by purposive searches—resulting also from engagement with stakeholders. Data were extracted from included articles and subjected to realist analysis to identify (i) mechanisms causing mental ill-health in doctors and medical students and relevant contexts or circumstances when these mechanisms were likely to be ‘triggered’ and (ii) ‘guiding principles’ and features underpinning the interventions and recommendations discussed mostly in policy document, reviews and commentaries.

**Results:**

One hundred seventy-nine records were included. Most were from the USA (45%) and were published since 2009 (74%). The analysis showed that doctors were more likely to experience mental ill-health when they felt isolated or unable to do their job and when they feared repercussions of help-seeking. Healthy staff were necessary for excellent patient care. Interventions emphasising relationships and belonging were more likely to promote wellbeing. Interventions creating a people-focussed working culture, balancing positive/negative performance and acknowledging positive/negative aspects of a medical career helped doctors to thrive. The way that interventions were implemented seemed critically important. Doctors and medical students needed to have confidence in an intervention for the intervention to be effective.

**Conclusions:**

Successful interventions to tackle doctors’ and students’ mental ill-health are likely to be multidimensional and multilevel and involve multiple stakeholders. Evaluating and improving existing interventions is likely to be more effective than developing new ones. Our evidence synthesis provides a basis on which to do this.

**Study registration:**

PROSPERO CRD42017069870. Research project webpage http://sites.exeter.ac.uk/cup/

## Background

The mental ill-health of doctors is a global, increasingly pressing and intractable problem [1–4] affecting all medical specialties and career stages [5, 6]. Its consequences are significant and far-reaching, both within and beyond healthcare, and include poor quality or inequitable patient care [7, 8], absenteeism (doctors taking short or long-term sick leave), presenteeism (doctors working whilst unwell), poor workforce retention (doctors leaving the profession temporarily/permanently) and even suicide [9]. The processes leading to mental ill-health of doctors and medical students are also complex and multiple, encompassing individual, group, organisational, professional and socio-cultural dimensions. Examples of these dimensions comprise the demanding nature of the profession, the pressure to deliver excellent care with shrinking resources, loss of autonomy and erosion of professional values, and perceived stigma around mental illness [10].

Most research to date is undertaken within disciplinary silos and fails to consider simultaneously the many dimensions that may negatively affect doctors’ wellbeing [11–13]. Notably, the current emphasis on resilience places responsibility for good mental health with the individual doctor, but resilience training alone is not likely to solve such a complex and multidimensional issue and may even aggravate how doctors experience work-related pressures, potentially contributing to mental ill-health [14]. We found evidence of this from both systematic reviews and commentaries [11–13, 15].

Some positive signs are starting to surface. An important recent example is the 11th revision of the International Classification of Diseases (ICD-11) which classifies burnout as an *occupational phenomenon* rather than a medical condition.^1^ This classification challenges the emphasis on resilience and individual responsibility for burnout, highlighting the role (and therefore the responsibility) of the workplace. There is also a growing interest in a range of resources and initiatives that promote doctors’ and medical students’ wellbeing—at different levels, e.g. preventive, screening and therapeutic—both locally [16] and internationally [15].

However, more needs to be done to prevent further deterioration in the mental ill-health of doctors and medical students. In the United Kingdom (UK), the wellbeing-related results from the 2018 annual NHS staff survey are the worst in the last 5 years, with almost 40% of respondents indicating they had been unwell in the past 12 months because of work-related stress [17, 18].

In order to drive positive change, it is vital for research to take an approach consistent with understanding the complexity of the problem. Doing so requires an interdisciplinary perspective that can bridge different viewpoints and approaches [19, 20]. By using an interpretive, theory-driven approach to analysing data from diverse literature sources, realist reviews move beyond description to provide findings that explain how and why contexts can influence outcomes, taking account of the many dimensions (e.g. individual, organisational, socio-cultural) of the problem and the heterogeneity of approaches and interventions [21]. By undertaking a realist review and by working in a multidisciplinary research team, we have been able to find, analyse and synthesise research from across disciplinary and professional silos and engage stakeholders in understanding what our findings mean in practice for driving forward change in the system. This is the first ever realist review of interventions to tackle doctors’ mental ill-health and its impacts on the clinical workforce. Through this novel approach to analysis, we have been able to generate new insights which have allowed us to make different kinds of recommendations and interventional strategies than other types of research could do.

## Methods

### Aim and objectives

The aim of our research was to improve understanding of how, why and in what contexts mental health services and support interventions can be designed in order to minimise the incidence of doctors’ and medical students’ mental ill-health. The main objectives were to:

1. Conduct a realist review on interventions to tackle doctors’ and medical students’ mental ill-health and its impacts on the clinical workforce and patient care, drawing on a wide range of literature sources and engaging iteratively with diverse stakeholder perspectives to produce actionable theory.
2. Produce recommendations that support the tailoring, implementation, monitoring and evaluation of contextually sensitive strategies to tackle mental ill-health and its impacts.

### Review process

The realist review followed a detailed protocol which has previously been published [10]. In this section, we provide a brief overview of our detailed review protocol. Our realist review is based on Pawson’s five iterative stages: (1) locating existing theories, (2) searching for evidence, (3) selecting articles, (4) extracting and organising data and (5) synthesising the evidence and drawing conclusions [22]. The reporting and conduct of this review is consistent with the RAMESES publication standards and reporting for realist reviews respectively [23].

### Step 1: Locating existing theories

In this first step of the review, we built the initial programme theory (a set of theoretical explanations or assumptions about how a particular programme, process or interventions is expected to work) [22]. We identified theories that helped to (i) understand the processes leading to mental ill-health in doctors and (ii) understand how interventions aiming to support doctors experiencing mental ill-health are supposed to work (and for whom), when they work, when they do not, why they are not effective and why they are not being used. We iteratively drew on informal iterative discussions, advice and feedback from key content experts representing multidisciplinary perspectives in our Stakeholder Group and an exploratory search of relevant literature.

### Step 2: Searching for evidence

Our search was designed, piloted and conducted by an information specialist (SB) in consultation with the co-authors. Search terms were derived from the titles, abstracts and indexing terms of relevant studies already known to the review team from background reading and consultation with stakeholders. These ‘empirically derived’ search terms were supplemented with relevant synonyms selected in consultation with the review team. Several versions of the search strategy were tested in MEDLINE via Ovid by checking that the relevant pre-identified studies were returned and by refining the search terms to optimise the relative recall and precision of the search (i.e. maximising the retrieval of known relevant studies whilst minimising the retrieval of irrelevant studies). In the process of testing and refining the search, we identified additional relevant studies which we also made sure were retrieved in subsequent iterations of the search (see full search strategy in Additional file 1). In December 2017, the final search strategy was translated and run in a selection of medical and psychology bibliographic databases, including MEDLINE, MEDLINE In-Process and Other Non-indexed Citations and PsycINFO (all via Ovid) and ASSIA (via ProQuest). Search results were exported to Endnote (X8, Clarivate Analytics, Philadelphia, USA) and de-duplicated using the automated deduplication feature and manual checking. We did not apply a historical date limit to the database searches. We were particularly interested in the UK setting; therefore, we supplemented bibliographic database searches with forward citation searching of included studies that were set in the UK.

### Step 3: Selecting articles

We included all sources that focussed on mental ill-health, absenteeism, presenteeism or workforce retention; all study designs; all healthcare settings; all studies that included medical doctors/medical students; descriptions of interventions or resources that focus on improving mental ill-health and minimising its impacts; and all mental health outcome measures, including absenteeism, presenteeism and workforce retention.

Using Endnote X8, DC screened the titles and abstracts of all articles resulting from the main and supplementary searches. A random 10% sample of the three sets of results were also screened independently for consistency of application of the inclusion criteria by CP (the second reviewer). Small inconsistencies were identified and resolved through discussion. DC then screened the full texts of the papers resulting from the first round of screening and classified them in categories based on their potential to contribute to programme theory.

The full text of a 10% sample of documents from the main search and a separate 10% sample of full texts from the supplementary searches were assessed and discussed between DC and CP to ensure that decisions for final inclusion and classification into categories have been made consistently. Small inconsistencies were identified that were resolved through discussion.

### Step 4: Extracting and organising data

The analysis was driven by a realist logic of analysis [24]. We sought to interpret and explain mechanisms (e.g. the way in which individuals and groups respond to and reason about the resources, opportunities or challenges offered by a particular intervention) causing mental ill-health in doctors and medical students and to identify relevant contexts when these mechanisms were likely to be ‘triggered’. These contexts and mechanisms and the resultant outcomes became the way we expressed our causal claims—i.e. we developed and expressed our causal claims in the form of context-mechanism-outcome configurations (CMOcs).

### Step 5: Synthesising the evidence and drawing conclusions

We compared and contrasted these CMOcs with the evolving programme theory, so as to understand the place of and relationships between each CMOc within the programme theory. As the review progressed, we iteratively refined the programme theory based on the analysis of data found within included sources, and by stakeholder feedback (see below). Our programme theory and the underpinning CMOcs enabled us to identify the interventions which were more likely to reduce or prevent mental ill-health in doctors, as well as important obstacles to the access and effectiveness of such interventions. We then developed guiding principles based on our programme theory, CMOcs and their related interventions.

### Stakeholder group

We engaged with a stakeholder group throughout the lifecycle of the project, from the development of the project grant to dissemination. Consultations with stakeholder group members took place as part of 2-h meetings at regular intervals—every 2 months—throughout the project (which lasted 18 months). We arranged additional face-to-face meetings with some stakeholders who could not attend the main meetings, as well as using Skype, email exchanges and/or telephone conversations. The group was originally composed of 22 people, including patient representatives, clinicians, doctors-in-training and medical educators, and expanded to include policy makers and members of professional bodies such as the General Medical Council and Health Education England in the latter stages. The group provided content expertise, contributed to refinement of our programme theory and is currently involved in the dissemination of the project’s non-academic outputs [10]. The meetings usually started with a brief slide presentation by our project team to introduce stakeholders to the topic under discussion and realist review approach and to provide a quick update on progress with the review. We presented our findings under development to the group in the form of statements and visual prompts to obtain their feedback. Discussions were designed to be more open-ended in the early stages of the review, but focussed on particular aspects of our results as the project progressed. Later stakeholder groups focussed on actionable findings and dissemination of the study. Additional file 2 provides a list of the face-to-face meetings, including the number of participants and key topics discussed.

## Results

**Fig. 1:**
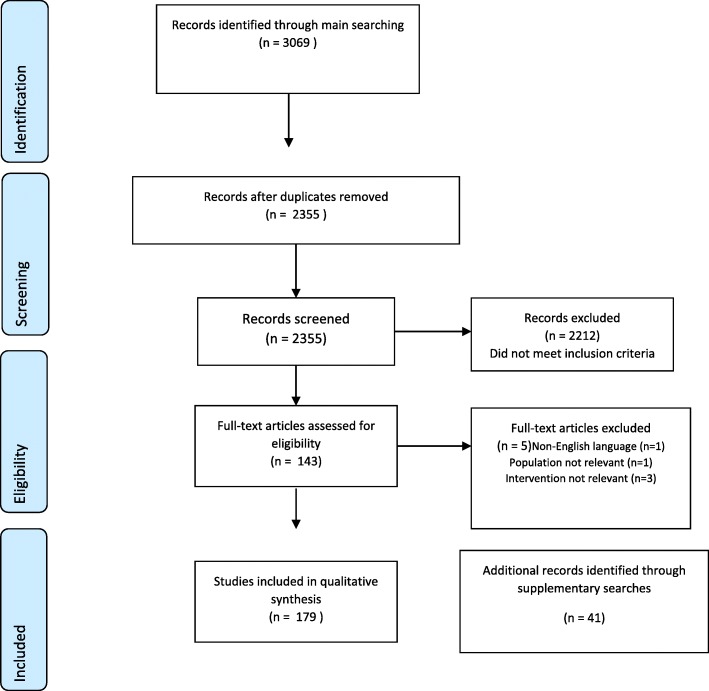
PRISMA diagram

The main searches identified 3069 studies; 179 studies met the inclusion criteria and were included in the review (see Fig. 1 for a PRISMA flowchart and Additional file 3 for a table describing the included studies). Of the included studies, the country most represented was the USA (45%) (22.3% of the included studies were from the UK); 74% had been published in 2010 or more recently; most were research studies; 33% focussed on structural interventions (those that require changes in the organisation of doctors’ work environment, e.g. rotas), 21% focussed on individual interventions (those that target the individual doctor, e.g. mindfulness) and 46% considered both levels together. Most interventional studies were preventative, rather than considering treatment or screening. Less than a quarter of included studies (19%) provided cost information. Of these, costs in 5/179 (3%) were quantified, 24/179 (13%) contained unquantified narrative claims and 6/179 (3%) contained a mix of quantified costs and unquantified narrative claims. No included studies reported a health economic analysis. Finally, most studies referred to doctors or physicians in general, rather than focussing on specific specialties or career stages.

Our realist analysis developed 19 CMOcs, structured around four main clusters: (1) processes leading to mental ill-health: isolation; (2) reducing mental ill-health: groups, belonging and relationality; (3) reducing mental ill-health: balance and timeliness; and (4) implementation methods: engendering trust. Table 1 provides a summary of the 19 CMOcs, which together make up the programme theory, organised in these four main clusters. The clusters are strongly intertwined, meaning that the likelihood of the success of an intervention strategy is enhanced by its alignment with all the four clusters. Examples of the citations that provide evidence for each CMOc are available in Additional file 4.

**Table 1:** Summary of the CMOcs (programme theory)

**(1) Processes leading to mental ill-health in doctors: isolation**	
CMOc 1: Underdeveloped workforce planning	In a workplace in which basic support structures to enable doctors to do their job are not in place (C), doctors may feel they must make up for the deficiencies of the organisation for patients and colleagues (M). This may contribute to a toxic working culture in which overwork and its negative consequences are normalised (O).
CMOc 2: Normalisation of high workload	When high workload and its negative consequences (e.g. distress, burnout) are normalised (C), overworked or sick doctors may feel they are letting down their colleagues and patients (M). This can contribute to presenteeism (O) and associated negative consequences on mental health (O1) and workforce retention (O2).
CMOc 3: Loss of autonomy	When doctors experience lack of autonomy over their work (C1), and some aspects of their work as less meaningful (C2), they may feel dissatisfied with their job (e.g. because they are unable to do the job they were trained for) (M). This can make doctors more vulnerable to stress and mental ill-health, irrespective of workload (O).
CMOc 4: Stigma towards vulnerability	In a professional culture where mental ill-health and vulnerability may be seen as unprofessional (C), doctors (and medical students) may feel ashamed (M1) or afraid (M2) of not living up to their professional identity if they experience mental ill-health (or other difficulties at work). This can lead doctors (and medical students) to adopt strategies which involve hiding their difficulties from themselves and colleagues (O).
CMOc 5: Hiding vulnerability	Where there is mental health support available for doctors (C1), doctors, who understand the system and that confidentiality is difficult to achieve (C2), may fear that seeking support could jeopardise their career (M) and so they may hide their distress rather than seek support (O).
CMOc 6: Isolation	When doctors work in physical and emotional isolation (C), they are likely to feel less supported by their colleagues and/or their employing organisation (M1) and/or mistrust of these groups (M2). This can make doctors more vulnerable to work-related pressure and mental ill-health (O).
**(2) Reducing mental ill-health: groups, belonging and relationality**	
CMOc 7: Positive and meaningful workplace relations	Positive and meaningful workplace relations (C) can foster a sense of belonging between colleagues and towards the medical profession (M). This can lead to an increased capacity to work under pressure (O)
CMOc 8: Functional working groups	Working in functional groups (C) can make doctors feel more supported (M1) and more at ease with vulnerability (M2). This can normalise vulnerability (O1) and reduce the stigma around mental ill-health (O2)
CMOc 9: Balancing quality and quantity of time at work	When doctors (for different reasons) have less connectedness and meaning at work (C), they may feel they can only find fulfilment outside work (M1), making it less likely that their condition will improve (O).
CMOc 10: Limits of groups	Sick doctors (and medical students) with particularly delicate circumstances (C) may not feel safe to share their problems (M1) and/or may not identify with the other group members (M2). This can result in a dysfunctional group (O1) and intensification of mental ill-health in doctors (O2).
CMOc 11: ‘Organic’ spaces to connect	If there are protected times and psychologically safe spaces for students/doctors to congregate within the confines of the work environment (C), students/doctors are likely to bond over whatever is most important to them at that time (M). This may improve connectedness (O).
**(3) Reducing mental ill-health: balance and timeliness**	
CMOc 12: Recognising both positive and negative performance	Where supervision and feedback recognise both positive and negative performance and promote doctors’ (and students’) learning from both of these (C), doctors (and students) may feel more fairly treated (M1) and more inclined to value their colleagues and employing organisation (M2), potentially leading to more connectedness and engagement at work (O1), and a more supportive work culture (O2).
CMOc 13: Balancing prevention of metal ill-health with promotion of wellbeing	In a work environment that actively demonstrates the importance of the balance between health and wellbeing with fighting stress and mental ill-health (C), doctors (and students) are more likely to feel that caring about their own wellbeing is legitimate (M1) and less afraid to acknowledge vulnerability (M2). This can contribute to a de-stigmatisation of mental ill-health and vulnerability (O).
CMOc 14: Acknowledging the positive and negative aspects of the profession	Where both the positive and negative aspects of a medical career are recognised (C), doctors (and medical students) may feel less inadequate and helpless when they or their colleagues experience stress or mental ill-health (M). This may lead to increased capacity to deal with work pressure (O1) and to recognition and acceptance of vulnerability (O2).
CMOc 15: Timely support	Timely support when doctors (and students) are particularly vulnerable (e.g. after a suicide attempt, death of a colleague, addiction) (C) may represent their only source of hope (M) and reduce the intensity of mental ill-health and its related outcomes, including suicide (O).
**(4) Implementation methods: engendering trust**	
CMOc 16: Endorsement	Doctors are less likely to engage with an intervention (O) if it is not endorsed by the employing organisation and senior leadership (C) because they may then lack trust in it (M1) and may also feel frustrated (M2) if they cannot access it due to work constraints.
CMOc 17: Expertise	If those delivering interventions do not have specific training to address the needs of sick doctors (C), the recipients may be less likely to trust the intervention (M) and the intervention may be ineffective (O1) and/or harmful (O2) or not accessed at all (O3).
CMOc 18: Engagement	If doctors (and students) are involved in the development and implementation of interventions (C), the recipients are more likely to trust (M1) and feel ownership (M2) of the intervention. As a result, it is more likely to be used (O1) and to be effective (O2).
CMOc 19: Evaluation	If the outcomes of interventions and the wellbeing of the workforce are regularly reviewed and monitored (C1), and commitment to act upon the outcome of these regular review exercises is shown (C2, and CMOc 16), then doctors may feel more supported (M) and engage with efforts to tailor these interventions (O1). This may also lead to greater awareness about vulnerability and wellbeing in the workplace (O2).

### Processes leading to mental ill-health: isolation

Our analysis revealed that doctors (and medical students) are more likely to experience mental ill-health when they feel isolated, when they feel unable to do the job they were trained for and when they fear the repercussions of seeking help and support (CMOcs 1–6). These processes blend organisational and work culture factors. The main organisational ones are resources and workload, work structure, workforce planning and governance (CMOcs 1–3). The main factors relating to work culture are medical culture and identity, and in particular the ideas of invulnerability, perfectionism and stigma around mental ill-health within the medical profession (CMOcs 4–6). Considerations of biographical aspects, related to individual psychological predisposition to mental ill-health, personal contexts and/or traumatic events outside of work, were often strongly intertwined with the dimensions described above.

### Reducing mental ill-health: groups, belonging and relationality

Our analysis also showed that interventions that emphasise relationships and belonging (for example with colleagues, to a healthcare team or the profession) are more likely to promote wellbeing and improve workplace cultures (CMOcs 7–11). The sense of belonging and connectedness, fostered by positive and meaningful workplace relations, can lead to an increased capacity to work under pressure (CMOc 7), can lead to an increased sense of meaning at work (CMOc 9) and can also contribute to normalise vulnerability and mental ill-health (CMOc 8). It is beyond the scope of this review to provide details of what is needed for initiatives to foster effective relationality, but, as suggested by some of our included sources (e.g. [25]) and research in psychology of health [26], relationships that are not imposed via mandatory activities and occur more spontaneously are particularly positive—for example, where the work environment provides protected time and safe spaces for student/doctors to congregate (CMOc 11). Our analysis also suggested that there are some limitations to group approaches and one-to-one support approaches are sometimes preferable, for example when there are significant confidentiality issues, when the individual is under investigation, or after the death of a colleague (CMOc 10).

### Reducing mental ill-health: balance and timeliness

Our analysis highlighted that interventions that create a people-focussed working culture which recognises that the health and wellbeing of staff is important both as a value in itself and as a necessary precondition to excellent patient care, promotes doctors and medical students learning from both positive and negative performance and acknowledges both the rewarding and stressful aspects of a medical career can help doctors to thrive at work and deal with work pressures (CMOCs 12–15).

It is common for pressured healthcare services to focus more on workforce mistakes, errors and negative patient outcomes and less on positive practice and performance [27, 28]. However, promoting supervision and feedback that recognise *both* positive and negative performance and encourage doctors and students to learn from both can lead to more connectedness and engagement in the workplace and to a more supportive work culture (CMOc 12). Similarly, a work culture that promotes health and wellbeing alongside focussing on fighting mental ill-health can contribute to the normalisation and de-stigmatisation of mental ill-health (CMOc 13). Acknowledging both rewarding and stressful aspects of the medical profession can also help to achieve the same outcomes as it may make students and doctors feel less inadequate when they or their colleagues experience mental ill-health or difficulties at work (CMOc 14).

The timeliness of support, especially when doctors or students are particularly vulnerable (e.g. in relation to traumatic events such as a suicide attempt, addiction and sickness absence), may reduce mental ill-health outcomes, including suicide (CMOc 15).

### Implementation methods: engendering trust

Our analysis suggests that for an intervention to be successful, it is important to consider not only its content but also how such intervention is implemented: the *how* is as important as the *what*. Doctors (and medical students) need to have confidence in an intervention, and those delivering it, for the intervention to be effective (CMOCs 16–19). Interventions are more likely to work if they are endorsed by the senior level of the organisation involved (CMOc 16), those delivering them have adequate training to address the needs of sick doctors’ and students’ (CMOc 17), doctors (and students) are engaged in their design (CMOc 18) and the outcomes of such interventions are regularly reviewed and effectively acted upon (CMOc 19).

## Discussion

The aim of our realist review was to improve understanding of how, why and in what contexts mental health services and support interventions can be designed in order to minimise the incidence of doctors’ and medical students’ mental ill-health. Our review identified three important processes leading to mental ill-health: significant and complex workload, organisational management and the professional culture of medicine.

Working relationships, with other doctors and healthcare staff as well as with the organisation, explained a key part of how mental ill-health could be prevented and reduced. Whilst not operating in a linear fashion, meaningful workplace relations and functional working groups are likely to enable the protective mechanisms of a *sense of belonging* and an *ease with vulnerability* to function. Moreover, the, often implicit, vision set by healthcare organisations and professional bodies about what a medical career entails and how different clinicians are expected to behave could have important implications for mental ill-health. Constructive workplace feedback on performance, an organisation’s demonstrable valuing of employees’ health and wellbeing and a professional culture that recognises both the rewarding and stressful aspects of a medical career could provide the context for doctors (and students) to contribute to and benefit from an upward cycle of interacting mechanisms such as legitimating their own wellbeing, acknowledging vulnerability, preventing feelings of inadequacy and valuing colleagues. Finally, when doctors and students are affected negatively by significant life events, the provision of timely support in the workplace is crucial for hope to play a role in recovery.

Whilst interventions that target individual doctors and students (e.g. to prevent mental ill-health or ameliorate it once established) may address parts of the processes leading to mental ill-health, interventions that address multiple organisational and professional issues *simultaneously* are more likely to be successful.

By undertaking a realist review, we have been able to find, analyse and synthesise data from across disciplinary silos and ‘sense check’ our findings against feedback from various perspectives. Our multidisciplinary research team worked closely with a large, diverse group of individuals in the stakeholder group and sense-making groups and ‘translated’ the findings through these interactions. This was in contrast to much of the research into doctors’ mental ill-health identified through our review, which tended to be limited to quite specific perspectives occurring in disciplinary silos [11–13, 15]. An important limit of this approach is the lack of consideration of the organisational and structural contexts that may have a detrimental effect on doctors’ wellbeing. There are however commentators who have argued that interventions should *also* focus on organisational support and systemic factors contributing to mental ill-health, rather than on individual doctors [9, 29, 30]—some have advocated for a shift from ‘individual’ to ‘organisational resilience’ [13]. Our study corroborates this multidimensional approach.

The key limitation of our research, in common with all literature-based research methods, is that the findings are only as good as the literature available to synthesise. Most studies about individual- and work-related factors related to doctors’ mental ill-health are of a cross-sectional design; however, it is still epistemologically possible to make causal claims based on such data, especially by combining data of various types, as in a realist review [21, 24]. In this review, most of the literature came from the USA, with a smaller component from the UK, which made the interpretation of the relevance of the data from included sources to the UK healthcare setting more challenging. We were assisted through the methodological challenges related to the transferability of our findings to a UK setting by our stakeholder group.

## Conclusions

Drawing on our analysis and engagement with key stakeholders, we articulated recommendations and implementation principles about intervention strategy design and development for different audiences (Table 2). Whilst these recommendations and principles are presented separately to aid comprehension, the interdependency of these levels should be acknowledged in intervention strategy design and development. For example, doctors may not be able to prioritise relationships at work if there are no organisational structures to support this. To maximise the impact of our review and help those refining/designing interventional strategies to tackle doctors’ mental ill-health, we also developed 10 ‘Care Under Pressure’ principles, by which existing interventions might be refined, which draw out the interdependence of the different levels (Table 3).

**Table 2:** Key recommendations and principles for refining/developing strategies to reduce mental ill-health

**Key recommendations and principles for refining/designing strategies to reduce mental ill-health in doctors**	
For policy makers	Policies that aim to secure the future of the NHS workforce must foster a supportive work culture in which individuals can thrive. Policies and interventions that target the individual in the absence of a supportive work culture are unlikely to succeed. CMOCs 1–3, 7–9, 12–14, 16, 19.
For employers	Ensure influential nominated Board-level responsibility for the wellbeing of staff. This should include regular immersion in practice settings, as well as regular reports on progress against key performance indicators (e.g. absenteeism might be detected by sickness absence, rota gaps and vacant posts; presenteeism might be detected by complaints and errors; workforce retention might be detected by staff turnover; general staff wellbeing might be detected via annual staff surveys, markers of overwork and occupational health referrals). CMOCs 12–13, 16–19.
For team leaders	Actively look out for behaviours that may be potentially stigmatising and encourage help-seeking. In performance reviews, emphasise the positive as well as the negative and ensure the doctor knows their hard work in often challenging circumstances is valued. Make clear that prioritising own health is important for patient care. CMOCs 12–15.
For doctors	Recognise when you are working under pressure and, even when your workload is high, prioritise your relationships at work. CMOCs 7–11.
For other healthcare team members	Recognise that the whole team may, at times, be providing care under pressure. Try to normalise discussions of struggle in the context of challenging work. CMOCs 7, 8, 11–13.
For patients	Know that doctors and other health professionals are usually doing the best job they can in difficult circumstances. A thank you when things go well will always be appreciated! CMOCs 4, 5, 7, 12.
For researchers	Use research syntheses and stakeholder involvement to target your research to the areas of greatest need. Research of all kinds will be needed to develop theory and interventions, and design appropriate outcome measures, approaches to evaluation and implementation, in relation to doctors’ mental ill-health. CMOCs 1–19.
For those refining/designing interventions	Adopt our 10 Care Under Pressure principles (see below). CMOCs 1–19.

**Table 3:** Principles for use for those refining/designing interventional strategies to tackle doctors’ mental ill-health

**10 Care Under Pressure principles, for use by those refining/designing interventional strategies to tackle doctor mental ill-health**	
1. Be clear about who the intervention is for (given the continuum from full health, to ‘under pressure’, to mental ill-health).	
2. Give options by signposting to a range of interventions (e.g. a ‘one stop shop’ of local, regional and national resources).	
3. Ensure that information about the intervention is readily and rapidly available.	
4. Ensure that interventions are accessible to someone who works long and inflexible hours.	
5. At the initial enquiry stage, invest time in building trust and normalising stigma and struggle.	
6. Provide interventions in groups whenever possible, to prioritise connectedness, relationships and belonging.	
7. Ensure interventions for individuals are endorsed by or embedded in the workplace, where possible.	
8. Encourage and empower individuals to tackle low-level everyday hassles at work, to free up capacity to deal with bigger issues.	
9. Emphasise that prioritising and investing in physical and mental health is essential for optimal patient care.	
10. Evaluate and improve the intervention regularly, using data such as numbers and types of attendee, programme adherence and user perceptions.	

**Fig. 2:**
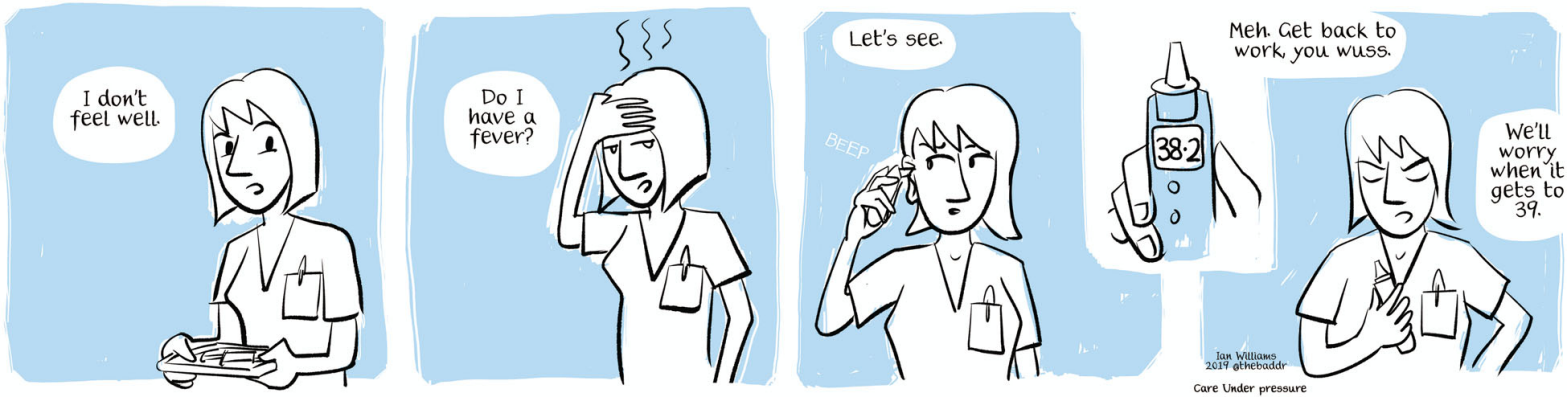
Example of Care Under Pressure creative output: a cartoon about self-care and presenteeism drawn by our collaborator Dr. Ian Williams—for more cartoons and information, please visit http://sites.exeter.ac.uk/cup/cartoons/

To support and bring to life our recommendations and principles, we have developed, with our stakeholders and with two artists and a film maker, a series of graphic illustrations and videos (see Fig. 2 for an example of cartoon about self-care and presenteeism^2^). These have been designed to surface the issues that underpin some of the recommendations and principles—making them more human and accessible.

A significant number of interventions are described in the peer-reviewed and grey literature, but many fall short of meeting the ‘10 Care Under Principles’ that we have developed through this research. Therefore, evaluating and improving existing interventions is likely to be more effective than developing new ones. The role of undergraduate medical education in forming doctors’ professional culture is important for the way it can develop healthy or unhealthy attitudes and behaviours in relation to coping and disclosure. If supportive working cultures can be created and sustained, medical schools could provide an early opportunity to proactively emphasise the importance of looking after one’s own health, to normalise discussions of struggle when work is challenging, to recognise the positive and negative aspects of medical careers and to promote an understanding of how and when to seek help.

Future research can build on our realist review to evaluate and refine interventional strategies that have been already implemented, or design, implement and evaluate new ones. For example, the Royal College of Surgeons and Health Education England are piloting a ‘modern firm’, one of whose main objectives is to make trainees ‘feel more valued while also recognising the needs of senior doctors: specifically their own need for training and for time to train and support their junior colleagues’ [31]. Such evaluations might use a realist evaluation approach or a complex intervention trial approach.

## Supplementary information

**Additional file 1.** Search 1: Interventions to reduce mental ill health.

**Additional file 2.** Table describing the stakeholder group meetings.

**Additional file 3.** Table describing the included studies.

**Additional file 4.** Examples of the citations that provide evidence for each CMOc.

## Data Availability

The authors will share the secondary data identified by this research.

## Abbreviations

CMOcs: Context-Mechanism-Outcome configurations
ICD: International Classification of Diseases

## Footnotes

1 For example, https://www.who.int/mental_health/evidence/burn-out/en/#.XO2TlgH2dTs.twitter

2 For more information, please visit http://sites.exeter.ac.uk/cup/.
