# Supplementary material for: Optimising strategies to address mental ill-health in doctors and medical students: ‘Care Under Pressure’ realist review and implementation guidance
Source: BMC Med. 2020 Apr 8;18:76. doi: 10.1186/s12916-020-01532-x (PMC7106831; doi:10.1186/s12916-020-01532-x)
Supplement: Supplementary file 1 — Additional file 1. Search 1: Interventions to reduce mental ill health. [file 12916_2020_1532_MOESM1_ESM.docx]

Search 1: Interventions to reduce mental ill health

Database: MEDLINE

Host: Ovid

Data Parameters: 1946 to November Week 4 2017

Date Searched: 6/12/2017

Searcher: SB

Hits: 1517

Strategy:

1. (doctor* or medic or medics or "medical trainee*" or "medical student*" or physician*).tw.
2. exp *Physicians/
3. *Students, Medical/
4. or/1-3
5. ("mental health" or "mental ill health" or stress* or distress* or anxiety or anxious or depression or depressed or "well being" or wellbeing or resilienc*).tw.
6. (pressure* adj3 (work* or "patient* demand*")).tw.
7. *Mental Health/
8. Stress, Psychological/
9. *Depression/
10. *Anxiety/
11. or/5-10
12. (retention or presenteeism or absenteeism or "leaving medicine" or "sick leave" or burnout or "burn* out").tw.
13. Presenteeism/
14. *Absenteeism/
15. *Sick Leave/
16. or/12-15
17. 4 and 11 and 16

Database: MEDLINE In-Process & Other Non-Indexed Citations

Host: Ovid

Data Parameters: 1946 to 6^th^ December 2017

Date Searched: 6/12/2017

Searcher: SB

Hits: 267

Strategy:

1. (doctor* or medic or medics or "medical trainee*" or "medical student*" or physician*).tw.
2. ("mental health" or "mental ill health" or stress* or distress* or anxiety or anxious or depression or depressed or "well being" or wellbeing or resilienc*).tw.
3. (pressure* adj3 (work* or "patient* demand*")).tw.
4. 2 or 3
5. (retention or presenteeism or absenteeism or "leaving medicine" or "sick leave" or burnout or "burn* out").tw.
6. 1 and 4 and 5

Database: PsycINFO

Host: Ovid

Data Parameters: 1806 to November Week 2 2017

Date Searched: 6/12/2017

Searcher: SB

Hits: 1025

Strategy:

1. (doctor* or medic or medics or "medical trainee*" or "medical student*" or physician*).tw.
2. exp *physicians/
3. *medical students/
4. or/1-3
5. ("mental health" or "mental ill health" or stress* or distress* or anxiety or anxious or depression or depressed or "well being" or wellbeing or resilienc*).tw.
6. (pressure* adj3 (work* or "patient* demand*")).tw.
7. *mental health/
8. psychological stress/
9. *major depression/
10. *anxiety/
11. or/5-10
12. (retention or presenteeism or absenteeism or "leaving medicine" or "sick leave" or burnout or "burn* out").tw.
13. *employee absenteeism/
14. *employee leave benefits/
15. or/12-14
16. 4 and 11 and 15

Database: ASSIA

Host: ProQuest

Data Parameters: n/a

Date Searched: 6/12/2017

Searcher: SB

Hits: 260

Strategy:

1. (ti,ab(doctor* OR medic or medics OR "medical trainee*" OR "medical student*" OR physician*) OR MAINSUBJECT.EXACT.EXPLODE("Doctors") OR MAINSUBJECT.EXACT("Medical students"))
2. (ti,ab("mental health" OR "mental ill health" OR stress* OR distress* OR anxiety or anxious OR depression OR depressed OR "well being" OR wellbeing OR resilienc*) OR (pressure* n/2 (work* OR "patient* demand*")) OR MAINSUBJECT.EXACT("Mental health") OR MAINSUBJECT.EXACT.EXPLODE("Stress") OR MAINSUBJECT.EXACT("Depression") OR MAINSUBJECT.EXACT("Anxiety") OR MAINSUBJECT.EXACT("Anxiety-Depression"))
3. (ti,ab(retention OR presenteeism OR absenteeism OR "leaving medicine" OR "sick leave" OR burnout OR "burn* out") OR MAINSUBJECT.EXACT("Absenteeism") OR MAINSUBJECT.EXACT.EXPLODE("Sick leave"))
4. 1 AND 2 AND 3

**Number of results per bibliographic database and in total**

| **Database** | **Number of results** |
| --- | --- |
| MEDLINE | 1517 |
| MEDLINE In-Process | 267 |
| PyscINFO | 1025 |
| ASSIA | 260 |
| **Total number of results** | **3069** |
| **Duplicate results** | **634** |
| **Total number of unique results** | **2435** |
