# Supplementary material for: Optimising strategies to address mental ill-health in doctors and medical students: ‘Care Under Pressure’ realist review and implementation guidance
Source: BMC Med. 2020 Apr 8;18:76. doi: 10.1186/s12916-020-01532-x (PMC7106831; doi:10.1186/s12916-020-01532-x)
Supplement: Supplementary file 2 — Additional file 2. Table describing the stakeholder group meetings. [file 12916_2020_1532_MOESM2_ESM.docx]

| **Date** | **Stakeholder group members** | **Key topics discussed** | **Examples of stakeholders’ contribution** |
| --- | --- | --- | --- |
| 30^th^Jan 2018 | Four patient representatives  Four clinicians  Three academics | Discussed inconsistencies in current support for doctors in the NHS, and the complexity of embedding intervention particularly in the current overworked context of doctors | - Doctors may not necessarily access services and support for fear of career repercussions and stigma  -Doctors identity also is influenced by patient expectations  - Need for ‘safe places’ for doctors: infrastructural, relational etc. (e.g. doctors’ mess) |
| 21^st^ Feb 2018 (satellite meeting) | Two medical educators, one of whom was a clinician One trainee | Discussed the support available to medical students across the UK, and the impact of medical school to doctoring culture | - Medical schools may take too long to refer sick students to external support  - There seems to be less support available after graduation |
| 13^th^ March 2018 | Three patient representatives  Four clinicians One academic | Discussed initial findings from the literature review and explored how these reflected stakeholders’ experiences | - Employers may be keener to engage with interventions if driven by a ‘business case’ rather than a ‘humanity case’  -Psychological and financial complexity of sickness absence pathways within the NHS  - The dilemma over making patients/public aware that doctors are human. It may help to destigmatise mental ill-health, but doctors themselves may not feel comfortable with this (they may not want patients to worry about their health and wellbeing) |
| 21 May 2018 | Three clinicians and academics | Discussed initial findings for the literature review and explored how these reflected stakeholder’s experiences | -The importance relationships and belonging for doctors wellbeing |
| 11^th^ September 2018 | Three patient representatives  Two academics One representative from relevant medical organisation Two clinicians | Continued the discussion of emerging findings from the review. Assessed stakeholder’s views on who is responsible for doctors’ wellbeing at work, and whether and how they would like to be involved with the production and disseminations of the project findings | - If support is implemented, the employer has to give time to doctors to engage with it. e.g. GPs cannot protect their lunch break as they are requested to be available in case of emergencies  - Balint or similar groups apart from having a function of increasing ‘resilience’ of doctors can also provide opportunity for professional learning (sharing best practice etc.)  - Difficulty with identifying responsibility for doctors’ wellbeing. Importance of giving doctors the right pathway into help when a crisis occurs |
| 8^th^ of October 2018 (satellite meeting) | One medical educator and clinician  One clinician and comics artist | Discussed initial findings from the literature review, explored how these reflected stakeholders’ experiences, discussed their willingness to be involved in co-production of dissemination output | - It can be challenging for teams to influence broader cultural changes in the work environment  - There is lack of attention towards sleep deprivation and fatigue of doctors (especially trainees). Sleep policy for trainees need to improve |
| 27^th^ February 2019 | One clinician  One clinician and academic One medical educator Three academics | Discussed the findings, how to convey them in an accessible way and the non-academic outputs | -Importance of linking doctors’ wellbeing and positive patient outcomes |
| 12^th^ March 2019 | Two medical educators and clinicians One medical educator One clinician Two patient representatives One researcher | Discussed the findings, the dissemination strategy for the non-academic outputs, and the stakeholders’ willingness to continue to collaborate in the production of these outputs | - Need to debunk the ‘mythology’ within the medical culture whereby mental ill-health and vulnerability is a taboo in doctors- from medical school onwards.  - Discussed examples of existing videos which de-stigmatise metal ill-health among doctors  - Discussed the importance of recognising the spectrum of mental ill-health e.g. distinguishing between acute and chronic degree of illness.  - Discussed the challenge of how to make some of our recommendations more meaningful to policy makers e.g. make the link between wellbeing of doctors and patient outcomes very clear |
