# Supplementary material for: Optimising strategies to address mental ill-health in doctors and medical students: ‘Care Under Pressure’ realist review and implementation guidance
Source: BMC Med. 2020 Apr 8;18:76. doi: 10.1186/s12916-020-01532-x (PMC7106831; doi:10.1186/s12916-020-01532-x)
Supplement: Supplementary file 3 — Additional file 3. Table describing the included studies. [file 12916_2020_1532_MOESM3_ESM.docx]

| Author | **Year** | **Country** | **Pub Type** | | **Method** | **Intervention Level** | **P** | **S** | **T** | **Target** | **Career stage** |
| --- | --- | --- | --- | --- | --- | --- | --- | --- | --- | --- | --- |
| Allen et al [1] | 2017 | Australia | | A | Quant | Structural | x |  | x | Obstetrics,  gynaecology | CT |
| Bakker et al [2] | 2000 | Netherlands | | B | Quant | Mixed | x |  |  | GPs | CT |
| Barbosa et al [3] | 2013 | USA | | A | Quant | Structural | x |  |  | Medical students | U |
| Bar-Sela, et al [4] | 2012 | Israel | | A | Quant | Structural | x |  |  | oncologists | TC |
| Beckman [5] | 2015 | USA | | C |  | Mixed | x |  | x | Primary care | TC U |
| Benson, Magraith[6] | 2005 | Australia | | C |  | Mixed | x |  |  | GP | C |
| Bitonte, DeSanto [7] | 2014 | USA | | C |  | Mixed | x |  |  | Medical students | U |
| Blais et al [8] | 2010 | Canada | | B | Quant | Mixed |  |  | x | Physicians | TC |
| Boorman [9] | 2009 | UK | | D |  | Mixed | x | x | x | NHS workforce | CT |
| Bragard et al [10] | 2006 | Belgium | | C |  | Mixed | x |  |  | Oncologists | TC |
| Brazeau [11] | 2010 | USA | | C |  | Structural | x |  |  | Medical students | U |
| Bugaj et al [12] | 2016 | Germany | | A | Quant | Structural | x |  |  | Medical students | U |
| Bugaj et al [13] | 2016 | Germany | | B | Review | Mixed: | x |  | x | Medical students | U |
| Burghi et al [14] | 2017 | USA | | A | Quant | Individual |  | x |  | 1^st^ year medical students | U |
| Carter et al [15] | 2005 | UK | | C |  | Mixed | x | x |  | Medial students | U |
| Carvour et al [16] | 2016 | USA | | B | Review | Structural |  |  | x | medical trainees | T |
| Chambers et al [17] | 2017 | New Zealand | | B | Mix | Structural | x |  |  | doctros and dentists | C |
| Chang et al [18] | 2013 | USA | | B | Quant | Individual |  |  | x | Medical students | U |
| Chaukos et al [19] | 2018 | USA | | B | Quant | Mixed | x |  |  | Residents | TC |
| Clarke et al [20] | 2014 | UK | | B | Qual | Mixed |  |  |  | Doctors | CT |
| Clough et al [21] | 2017 | Australia | | B | Systematic review | Mixed | x |  | x | Doctors | CT |
| Cockerell [22, 23] | 2016 Part I; 2017 Part II | USA | | C |  | Individual | x |  | x | Physicians | TC |
| Cornelius et al [24] | 2017 | USA | | A | Quant | Structural | x |  |  | Emergency medicine residents | TC |
| Cornwell et al [25] | 2017 | UK | | D |  | Mixed |  |  |  | NHS staff | CT |
| Davies et al [26] | 2016 | UK | | A | Quant | Individual |  |  | x | Doctors | CT |
| De Vibe et al [27] | 2013 | Norway | | A | RCT | Individual | x |  |  | Medical and psychology students | U |
| Deng et al [28] | 2016 | China | | B | Quant | Individual |  | x |  | Oncology doctors and nurses | CT |
| Department of Health [29] | 2008 | UK | | D | Review | Mixed | x | x | x | NHS doctors | CTU |
| Department of Health [30] | 2009 | UK | | D |  | Mixed | x | x | x | NHS workforce | CT |
| Devi [31] | 2011 | US | | B | Review | Mixed | x |  |  | Doctors and medical students | CTU |
| Dobkin, Hutchinson [32] | 2013 | Canada | | B | Literature review | Mixed | x |  |  | Medical students | U |
| Doran et al [33] | 2016 | UK | | B | Mix | Structural: | x |  |  | GP | C |
| Downs et al [34] | 2014 | USA | | B | Quant | Mixed: | x | x | x | Medical students | U |
| Dunn et al [35] | 2007 | USA | | A | Quant | Mixed: | x | x | x | Physicians | CT |
| Dyer [36] | 2018 | UK | | C |  | Structural |  |  | x | Doctors | T |
| Dyrbye et al [37] | 2016 | USA | | A | RCT | Individual | x |  |  | Physicians | TC |
| Dyrbye et al [38] | 2017 | USA | | B | Quant | Individual | x |  |  | Medical students | U |
| Dyrbye et al [39] | 2017 | USA | | B | Quant | Structural | x |  |  | First-year medical students | U |
| Eisenstein [40] | 2018 | USA | | C |  | Mixed | x |  |  | Doctors | CTU |
| Epstein, Krasner [41] | 2013 | USA | | C |  | Mixed |  |  |  | Physicians –other HCPs | TC |
| Ey et al [42] | 2013 | USA | | B | Quant | Structural | x |  | x | Residents | T |
| Ey et al [43] | 2016 | USA | | A | Quant | Mixed | x | x | x | Medical trainees and faculty | UTC |
| Feld et al [44] | 2006 | UK | | A | Quant | Structural | x |  | x | Palliative care | TC |
| Firth-Cozens [45] | 2007 | UK | | B | Review | Mixed | x |  |  | Psychiatrists (and doctors ) | CT |
| Flowers [46] | 2005 | USA /UK | | B | Review | Mixed | x |  |  | Medical students | U |
| Foster et al [47] | 2012 | USA | | A | Quant | Mixed | x |  |  | Residents | TC |
| Fothergill et al [48] | 2004 | UK | | B | Systematic review | Mixed | x |  | x | Physiatrists | TC |
| Gardiner et al [49] | 2015 | USA | | B | Quant | Mixed: | x | x | x | Family medicine residents | TC |
| Gardiner et al [50] | 2013 | Australia | | A | Quasi-experimental | Mixed: |  |  | x | GPs | C |
| Garelick et al [51] | 2007 | UK | | B | Quant | Individual |  |  | x | Doctors | TC |
| Garside [52] | 1993 | Canada | | B | Review | Structural: | x |  | x | Physicians (mostly primary healthcare) | TC |
| Gazelle et al [53] | 2015 | USA | | C |  | Individual | x |  | x | Physicians | TC |
| Geller et al [54] | 2008 | USA | | A | Mix | Individual |  | x |  | Clinical genetics | TC |
| George et al [55] | 2012 | USA | | A | Qual | Structural | x |  |  | Year 1 medical students | U |
| Gerada [56] | 2018 | UK | | C |  | Mixed | x |  |  | Doctors | CT |
| Goldhagen et al [57] | 2015 | USA | | A | Quant | Individual | x |  |  | Resident physicians | TC |
| Goodman, Schorling [58] | 2012 | USA | | A | Quant | Mixed | x |  |  | Physicians | TC |
| Graham et al [59] | 2000 | UK | | B | Quant | Structural: | x |  |  | Doctors | C |
| Gregory [60] | 2015 | USA | | A | Quant | Structural | x |  | x | Primary care | TC |
| Gulen et al [61] | 2016 | Turkey | | B | Quant | Structural |  | x |  | Emergency medicine residents | TC |
| Gunasingam et al [62] | 2015 | Australia | | A | RCT | Structural | x |  | x | Junior doctors | TC |
| Haizlip et al [63] | 2012 | USA | | C |  | Mixed | x |  |  | Physicians and trainees | TC |
| Hamader, Noehammer [64] | 2013 | Austria | | B | Qual | Mixed | x |  |  | Medical students, occupational health and psychology | UTC |
| Haramati et al [65] | 2017 | USA | | C |  | Mixed | x | x | x | Doctors and medical students | TCU |
| Harrison et al [66] | 2014 | UK | | B | Qual | Mixed | x | x |  | Doctors | C |
| Haward et al [67] | 2003 | UK | | B | Quant | Structural | x |  |  | Cancer teams | CT |
| Hegenbarth [68] | 2011 | Switzerland | | C |  | Structural |  |  | x | Doctors | TCU |
| Hill et al [69] | 2016 | UK | | B | Systematic review | Mixed: | x |  | x | Palliative care staff | TC |
| Hill, Smith [70] | 2009 | USA | | B | Quant | Mixed: |  | x |  | Residents in academic otolaryngology | TC |
| Hlubocky et al ^[71]^ | 2016 | USA | | C |  | Mixed | x | x | x | Oncology | C |
| Hochberg et al [72] | 2013 | USA | | A | Quant | Structural: | x | x |  | Resident Surgeons | TC |
| Holoshitz , Wann [73] | 2017 | USA | | C |  | Individual: | x |  | x | Physicians | C |
| Horsfall [74] | 2014 | UK | | D |  | Mixed | x |  | x | Doctors under investigation | CT |
| Hotchkiss [75] | 2008 | USA | | B | Quant | Mixed: |  |  | x | Physicians | TC |
| Howlett et al [76] | 2015 | Canada | | A | Quant | Mixed | x |  |  | Emergency medicine staff |  |
| Ireland et al [77] | 2017 | Australia | | A | RCT | Individual: | x |  | x | Doctors | TC |
| Ro et al [78] | 2010 | Norway | | B | Quant | Individual |  |  | x | Physicians | TC |
| Ro et al [79] | 2012 | Norway | | B | Quant | Mixed | x |  | x | Doctors | TC |
| Kemper, Khirallah [80] | 2015 | USA | | A | Quant | Individual | x |  |  | Doctors and other health workers | CT |
| Kjeldmand , Holmström [81] | 2008 | Sweden | | B | Qual | Structural: | x |  |  | GPs | C |
| Kotter et al [82] | 2015 | Germany | | B | Qual | Structural | x |  | x | Medical students | U |
| Krasner et al [83] | 2009 | USA | | A | Quant | Individual: | x |  |  | Primary care | C |
| Kumar [84] | 2011 | New Zealand | | C |  | Structural: | x |  |  | Psychiatrists | CT |
| Kushnir et al [85] | 1994 | Israel | | A | Quant | Individual: | x |  |  | Occupational health | CT |
| Lapa et al [86] | 2016 | Portugal | | B | Quant | Mixed: |  | x |  | Anaesthesia | TC |
| Lederer et al [87] | 2008 | Austria | | B | Quant | Structural | x |  |  | Intensive care Physicians/ nurses | CT |
| Lee et al [88] | 2016 | Taiwan | | B | Quant | Mixed |  | x |  | Hospital staff | C |
| Lefevbre [89] | 2012 | Canada | | C |  | Mixed | x | x | x | Residents / physicians | TC |
| Leff et al [90] | 2017 | USA | | C |  | Structural | x |  | x | End of life care physicians and social workers | TC |
| Lim et al [91] | 2009 | New Zealand | | B | Quant | Structural | x |  |  | Radiologists | CT |
| Linzer et al [92] | 2015 | USA | | A | RCT | Structural | x |  | x | Primary care physicians | C |
| Linzer et al [93] | 2001 | USA and Netherlands | | B | Quant | Mixed | x |  |  | Physicians | TC |
| Luthar et al [94] | 2017 | USA | | A | RCT | Structural | x |  | x | Physicians | CT |
| Lyons et al [95] | 2017 | UK and Ireland | | C |  | Mixed | x |  | x |  | CTU |
| Mache et al [96] | 2017 | Germany | | A | Quasi-experimental | Mixed: | x |  |  | Oncology | CT |
| Maslach et al [97] | 2017 | USA | | B | Review | Mixed: | x |  | x |  | CTU |
| McCartney [98] | 2018 | UK | | C |  | Structural | x |  |  |  | CT |
| McClafferty , Brown [99] | 2014 | USA | | B | Review | Mixed |  |  |  | Physicians/ paediatricians | TC |
| McCray et al [100] | 2008 | USA | | B | Review | Mixed | x | x | x | Students-doctors- HCPs | UTC |
| McCue, Sachs [101] | 1991 | USA | | A | Quant | Mixed | x |  | x | Paediatrics residents | TC |
| McKenna et al [102] | 2016 | USA | | C |  | Structural | x |  |  | Medical students | U |
| McKevitt et al [103] | 1997 | UK | | B | Qual | Structural | x | x | x | Doctors | CTU |
| McKinley et al [104] | 2017 | USA | | B | Review | Mixed: | x | x | x | Paediatric residency | T |
| McManus et al[105] | 2011 | UK | | B | Quant | Individual | x |  |  | Doctors | TC |
| McNeill et al [106] | 2014 | Australia | | B | Quant | Structural | x |  |  | Medical students | U |
| Mechaber et al [107] | 2008 | USA | | B | Quant | Structural | x |  | x | Generalist Physicians | C |
| Merteen et al [108] | 2014 | UK | | B | Quant | Individual | x | x | x | Doctors | TC |
| Metha et al [109] | 2016 | USA | | A | Quant | Structural | x |  |  | Palliative care team | TC |
| Milstein et al [110] | 2009 | US | | A | Mixed | Individual |  | x | x | Paediatric house officers | T |
| Montgomery et al [111] | 2011 | Greece/UK | | B | Review | Mixed | x |  |  | Hospital staff | TC |
| Moutier et al [112] | 2012 | USA | | B | Quant | Structural | x | x |  | Medical students, residents, faculty | UTC |
| Murdoch et al [113] | 2007 | UK | | A | Quant | Structural | x |  | x | Psychiatrists | C |
| Myszkowski et al [114] | 2017 | France | | B | Quant | Individual |  | x |  | Internal medicine | TC |
| NHS England [115] | 2016 | UK | | D |  | Mixed |  |  |  | NHS workforce |  |
| Nielsen, Tulinius [116] | 2009 | Denmark | | B | Qual | Structural | x |  |  | 9 GPs | TC |
| Nomura et al [117] | 2016 | Japan | | B | Mix | Structural | x |  |  | Paediatric residents | TC |
| Oczkowski [118] | 2015 | Canada | | C |  | Mixed | x |  |  | Medical students/physicians | UTC |
| Oman et al [119] | 2006 | USA | | A | RCT | Individual | x |  | x | Doctros/HCPs | CT |
| Paice et al [120] | 2004 | UK | | B | Quant | Structural | x |  |  | Doctors in training | T |
| Paice, et al [121] | 2002 | UK | | B | Quant | Mixed | x | x | x | Doctors | C |
| Panagioti et al [122] | 2017 | UK | | B | Systematic review and meta-analysis | Mixed | x |  | x | Physicians | CT |
| Penfold [123] | 2018 | UK | | C |  | Structural: | x |  |  | Trainees | T |
| Pereira et al [124] | 2015 | Brazil | | B | Qual | Individual | x |  |  | Medical students | U |
| Prins et al [125] | 2007 | The Netherlands | | B | Quant | Structural | x |  | x | Residents | TC |
| Rabin et al [126] | 2005 | Israel | | C |  | Mixed | x | x | x | Doctors , mostly GP | TC |
| Raj [127] | 2016 | USA | | B | Systematic review | Mixed | x | x | x | Physicians/residents | TC |
| Ramirez et al [128] | 1996 | UK | | B | Quant | Structural | x |  |  | Gastroenterologists, surgeons, radiologists oncologists | CT |
| Ro et al [129] | 2016 | Norway | | B | Qual | Structural |  |  | x | Peer counsellors | CT |
| Regehr et al [130] | 2014 | Canada | | B | Review | Individual | x | x | x | Physicians | TC |
| Riley [131] | 2018a | UK | | B | Qual | Mixed | x |  |  | Mostly GPs | TC |
| Riley [132] | 2018b | UK | | B | Qual | Mixed | x |  | x | Mostly GPs | TC |
| Ringrose et al [133] | 2009 | The Netherlands | | A | Mix | Structural | x | x |  | Medical residents | T |
| Ripp et al [134] | 2016 | USA | | A | Quant | Structural | x |  | x | Residents | TC |
| Ripp et al [135] | 2017 | USA | | C |  | Mixed | x | x | x | Medical students | U |
| Ripp et al [136] | 2015 | USA | | B | Quant | Structural | x |  |  | Internal medicine residents | TC |
| Rippstein-Leuenberger et al [137] | 2017 | Switzerland | | B | Quant | Individual | x |  |  | Intensive care unit HCPs | CT |
| Ro et al [138] | 2008 | Norway | | A | Quant | Mixed |  |  | x | Doctors | C |
| Roberts et al [139] | 2002 | UK | | C |  | Structural | x | x | x | Psychiatrists | CTU |
| Robertson, et al [140] | 2010 | UK | | D |  | Mixed | x | x | x | NHS workforce |  |
| Rohland et al [141] | 2004 | USA | | A | Quant | Individual: |  | x |  | Physicians | TC |
| Rothemberger [142] | 2017 | USA | | B | Systematic review | Mixed | x |  | x | Physicians, medical students, trainees | CTU |
| Runyan et al [143] | 2016 | USA | | B | Quant | Structural | x |  |  | Family medicine residents | T |
| Sallon et al [144] | 2017 | Israel | | A | Quant | Mixed (mostly individual) | x |  | x | Hospital staff | CT |
| Sanchez et al [145] | 2016 | USA | | B | Mix | Structural |  |  | x | Physicians | TC |
| Schapira et al [146] | 2017 | Australia | | C |  | Mixed | x |  |  | Oncology | TC |
| Schattner [147] | 2017 | Israel | | C |  | Structural | x |  |  | Physicians | TC |
| Schmitz et al [148] | 2012 | USA | | C |  | Structural | x |  |  | Emergency medicine residents | TC |
| Schneider et al [149] | 2014 | USA | | B | Qual | Mixed | x |  | x | Physicians | TC |
| Scholz et al [150] | 2016 | Germany | | A | Quant | Individual | x |  |  | Medical students | U |
| Seoane et al [151] | 2016 | USA | | B | Quant | Structural | x |  |  | Medical students | U |
| Shanafelt et al [152] | 2012 | USA | | B | Quant | Individual | x |  |  | Surgeons | C |
| Shanafelt et al [153] | 2014 | USA | | A | Quant | Individual | x | x |  | Surgeons | C |
| Shapiro et al [154] | 2011 | USA | | B | Review | Individual | x |  |  | Physicians | C |
| Shapiro, Galowitz [155] | 2016 | USA | | C |  | Structural: | x |  | x | Doctors | TC |
| Sharifi [156] | 2012 | USA | | B |  | Individual | x |  | x | Mental health care providers | C |
| Shiralkar et al [157] | 2013 | USA | | B | Systematic review | Mixed: | x |  | x | Medical students | U |
| Siedsma, Emlet [158] | 2015 | USA | | A | RCT | Mixed | x | x | x | Physicians | TC |
| Sigsbee, Bernat [159] | 2014 | USA | | B | Review | Mixed | x |  | x | Physicians | TC |
| Slavin , Chinball [160] | 2016 | USA | | C |  | Mixed | x | x | x | Medical students, residents, physicians | UTC |
| Slavin et al [161] | 2011 | USA | | C |  | Mixed | x |  |  | Medical students /practicing physicians | UT |
| Smith [162] | 2016 | USA | | A | Action research | Structural | x |  | x | Medical students | U |
| Squiers et al [163] | 2017 | USA | | B | Review | Mixed |  |  |  | Physicians | TC |
| Talisman et al [164] | 2015 | USA | | B | Mix | Individual | x |  |  | Physicians, healthcare faculty | TC |
| Tucker et al [165] | 2017 | Canada | | A | Quant | Mixed | x |  |  | 3^rd^ year medical trainees | UT |
| Van Vilet et al [166] | 2017 | The Netherlands | | A | Quant | Mixed | x |  |  | Medical and nursing students | U |
| Verweij et al [167] | 2016 | The Netherlands | | A | Mix | Individual | x |  | x | GPs | C |
| Waddimba et al [168] | 2016 | USA | | B | Quant | Individual |  | x |  | Physicians | TC |
| Wald et al [169] | 2016 | USA/Israel | | A | Mix | Mixed | x |  |  | Medical, nursing faculty and medical students | UT |
| Warde et al [170] | 2014 | USA | | B | Quant | Mixed | x |  |  | Medical students | U |
| West et al [171] | 2014 | USA | | A | RCT | Structural | x | x |  | Physicians | TC |
| West et al [172] | 2016 | USA | | B | Systematic review and meta-analysis | Mixed | x | x | x | Physicians | TC |
| Wild et al [173] | 2014 | Germany | | B | Quant | Mixed: | x |  |  | Medical and psychology students | U |
| Wilkie et al [174] | 2005 | UK | | C |  | Structural | x |  | x |  | C T |
| Williams et al [175] | 1998 | UK | | D | Systematic literature review and interviews | Mixed | x | x | x |  | CT |
| Winefield et al [176] | 1998 | Australia | | A | Quant | Individual | x |  |  | GPs | TC |
| Wolf [177] | 1994 | USA | | B | Review |  | x |  |  | Medical students | U |
| Zhang et al [178] | 2017 | Canada | | A | Quant | Structural: | x | x | x | Trainee surgeons | CT |
| Zwack et al [179] | 2013 | Germany | | B | Qual | Mixed: | x |  | x | physicians | CT |

Table Legend

We classified the **publication types** as follows: **A** = Empirical research (intervention); **B** = Empirical research (non-intervention); **C** = Commentary (professional viewpoints based on experience); **D** = Policy. We classified the **methodology** used as follows: **Quant** = Quantitative; **RCT** = Randomised Controlled Trial; **Qual**= Qualitative; **Mix**= mixed methods. We classified the **levels of intervention** as follows: **Individual:** interventions that target the individual doctor. Typically: Mindfulness, cognitive behaviour techniques, (psycho/cognitive etc.) therapy, training to improve communication skills; **Structural:** Interventions that require changes in the organisation of doctors’ work environment. E.g. changes in schedule and reduction of workload, changes in operation of practice and healthcare organisation, group support, formal/ informal mentoring; **Mixed**: combination of the above E.g. blend of individual and structural, and individual intervention supported by organisational approaches. We classified the **level of intervention** as follows **P** = prevention; **S** = screening; **T** = therapy. Finally, we classified the **career stage** the included study targets/refers to as follows: **U**= medical student **T**=trainee; **C**= consultant

1. Allen, R., Watt, F., Jansen, B., Coghlan, E., Nathan, E.A., *Minimising compassion fatigue in obstetrics/gynaecology doctors: exploring an intervention for an occupational hazard.* Australasian Psychiatry, 2017. **25**(4): p. 403-406.

2. Bakker, A.B., Schaufeli, W.B., Sixma, H.J., Bosveld, W., van Dierendonck, D., *Patient demands, lack of reciprocity, and burnout: a five-year longitudinal study among general practitioners.* Journal of Organizational Behavior, 2000. **21**(4): p. 425-441.

3. Barbosa, P., Raymond, G., Zlotnick, C., Wilk, J., Toomey, R., 3rd, Mitchell, J., 3rd, *Mindfulness-based stress reduction training is associated with greater empathy and reduced anxiety for graduate healthcare students.* Education for health (Abingdon, England), 2013. **26**(1): p. 9-14.

4. Bar-Sela, G., Lulav-Grinwald, D., Mitnik, I., *"Balint group" meetings for oncology residents as a tool to improve therapeutic communication skills and reduce burnout level.* Journal of cancer education : the official journal of the American Association for Cancer Education, 2012. **27**(4): p. 786-9.

5. Beckman, H., *The role of medical culture in the journey to resilience.* Academic medicine : journal of the Association of American Medical Colleges, 2015. **90**(6): p. 710-2.

6. Benson, J., Magraith, K., *Compassion fatigue and burnout: the role of Balint groups.* Australian family physician, 2005. **34**(6): p. 497-8.

7. Bitonte, R.A., DeSanto, D.J., 2nd, *Mandatory physical exercise for the prevention of mental illness in medical students.* Mental Illness, 2014. **6**(2): p. 5549.

8. Blais, R., Safianyk, C., Magnan, A., Lapierre, A., *Physician, heal thyself: Survey of users of the Quebec Physicians Health Program.* Canadian family physician Medecin de famille canadien, 2010. **56**(10): p. e383-9.

9. Boorman, S., *The Final Report of the independent NHS Health and Well-being review,(2009). Department of Health.* NHS health and well-being review–the government response, 2009.

10. Bragard, I., Razavi, D., Marchal, S., et al., *Teaching communication and stress management skills to junior physicians dealing with cancer patients: a Belgian Interuniversity Curriculum.* Supportive care in cancer : official journal of the Multinational Association of Supportive Care in Cancer, 2006. **14**(5): p. 454-61.

11. Brazeau, C.M., *The surest way to prevent burnout in medical school and beyond.* Academic Medicine, 2010. **85**(4): p. No Pagination Specified.

12. Bugaj, T.J., Mucksch, C., Schmid, C., et al., *Peer-led Stress Prevention Seminars in the First Year of Medical School--A Project Report.* GMS journal for medical education, 2016. **33**(1): p. Doc3.

13. Bugaj, T., Cranz, A., Junne, F., Erschens, R., Herzog, W., Nikendei, C., *Psychosocial burden in medical students and specific prevention strategies.* Mental Health and Prevention, 2016. **4**(1): p. 24-30.

14. Bughi, S.A., Lie, D.A., Zia, S.K., Rosenthal, J., *Using a personality inventory to identify risk of distress and burnout among early stage medical students.* Education for Health, 2017. **30**(1): p. 26-30.

15. Carter, Y., Peile, E., *Selecting and supporting contented doctors.* British Medical Journal, 2005. **330**(7486): p. 269-270.

16. Carvour, M.L., Ayyar, B.K., Chien, K.S., Ramirez, N.C., Yamamoto, H., *A Patient-Centered Approach to Postgraduate Trainee Health and Wellness: An Applied Review and Health Care Delivery Model.* Academic medicine : journal of the Association of American Medical Colleges, 2016. **91**(9): p. 1205-10.

17. Chambers, C., Frampton, C., Barclay, M., *Presenteeism in the New Zealand senior medical workforce-a mixed-methods analysis.* The New Zealand medical journal, 2017. **130**(1449): p. 10-21.

18. Chang, E., Eddins-Folensbee, F., Porter, B., Coverdale, J., *Utilization of counseling services at one medical school.* Southern medical journal, 2013. **106**(8): p. 449-53.

19. Chaukos, D., Chad-Friedman, E., Mehta, D.H., et al., *Smart-r: A prospective cohort study of a resilience curriculum for residents by residents.* Academic Psychiatry, 2017: p. No Pagination Specified.

20. Clarke, R.T., Pitcher, A., Lambert, T.W., Goldacre, M.J., *UK doctors’ views on the implementation of the European Working Time Directive as applied to medical practice: a qualitative analysis.* BMJ open, 2014. **4**(2): p. e004390.

21. Clough, B.A., March, S., Chan, R.J., Casey, L.M., Phillips, R., Ireland, M.J., *Psychosocial interventions for managing occupational stress and burnout among medical doctors: a systematic review.* Systematic Reviews, 2017. **6**(1): p. 144.

22. Cockerell, C.J., *Pressure and disenchantment in physicians-Part I: Developing an approach to reconnect with what is noble about medicine.* Clinics in dermatology, 2016. **34**(5): p. 650-3.

23. Cockerell, C.J., *Pressure and disenchantment in physicians-Part II: Lessons for physicians from the Tao Te Ching.* Clinics in dermatology, 2017. **35**(1): p. 100-104.

24. Cornelius, A., Cornelius, B.G., Edens, M.A., *Increasing Resident Wellness Through a Novel Retreat Curriculum.* Cureus, 2017. **9**(7): p. e1524.

25. Cornwell, J., Fitzsimons, B., *Behind Closed Doors. Can we expect NHS staff to be the shock absorbers of a system under pressure?* 2017, The Point of Care Foundation.

26. Davies, S.R., Meerton, M., Rost, F., Garelick, A.I., *A sea change for sick doctors - how do doctors fare after presenting to a specialist psychotherapy service?* Journal of Mental Health, 2016. **25**(3): p. 238-244.

27. de Vibe, M., Solhaug, I., Tyssen, R., et al., *Mindfulness training for stress management: a randomised controlled study of medical and psychology students.* BMC medical education, 2013. **13**: p. 107.

28. Deng, Y.T., Liu, J., Zhang, J., et al., *A multicenter study on the validation of the Burnout Battery: a new visual analog scale to screen job burnout in oncology professionals.* Psycho-Oncology, 2017. **26**(8): p. 1120-1125.

29. Department of Health, *Mental health and ill health in doctors*. 2008: London, UK.

30. Department of Health, *NHS Health and Wellbeing Review: Interim Report*. 2009, The Stationary Office London.

31. Devi, S., *Doctors in distress.* The Lancet, 2011. **377**(9764): p. 454-455.

32. Dobkin, P.L., Hutchinson, T.A., *Teaching mindfulness in medical school: where are we now and where are we going?* Medical education, 2013. **47**(8): p. 768-79.

33. Doran, N., Fox, F., Rodham, K., Taylor, G., Harris, M., *Lost to the NHS: a mixed methods study of why GPs leave practice early in England.* The British journal of general practice : the journal of the Royal College of General Practitioners, 2016. **66**(643): p. e128-35.

34. Downs, N., Feng, W., Kirby, B., et al., *Listening to depression and suicide risk in medical students: the Healer Education Assessment and Referral (HEAR) Program.* Academic psychiatry : the journal of the American Association of Directors of Psychiatric Residency Training and the Association for Academic Psychiatry, 2014. **38**(5): p. 547-53.

35. Dunn, P.M., Arnetz, B.B., Christensen, J.F., Homer, L., *Meeting the imperative to improve physician well-being: assessment of an innovative program.* Journal of general internal medicine, 2007. **22**(11): p. 1544-52.

36. Dyer, C., *Julien Warshafsky: how this doctor died and what it tells us about the system that failed him.* BMJ, 2018. **361**: p. k2564.

37. Dyrbye, L.N., West, C.P., Richards, M.L., Ross, H.J., Satele, D., Shanafelt, T.D., *A randomized, controlled study of an online intervention to promote job satisfaction and well-being among physicians.* Burnout Research, 2016. **3**(3): p. 69-75.

38. Dyrbye, L.N., Satele, D., Shanafelt, T.D., *Healthy Exercise Habits Are Associated With Lower Risk of Burnout and Higher Quality of Life Among U.S. Medical Students.* Academic medicine : journal of the Association of American Medical Colleges, 2017. **92**(7): p. 1006-1011.

39. Dyrbye, L.N., Shanafelt, T.D., Werner, L., Sood, A., Satele, D., Wolanskyj, A.P., *The Impact of a Required Longitudinal Stress Management and Resilience Training Course for First-Year Medical Students.* Journal of General Internal Medicine, 2017. **32**(12): p. 1309-1314.

40. Eisenstein, L., *To Fight Burnout, Organize.* New England Journal of Medicine, 2018. **379**(6): p. 509-511.

41. Epstein, R.M., Krasner, M.S., *Physician resilience: what it means, why it matters, and how to promote it.* Academic medicine : journal of the Association of American Medical Colleges, 2013. **88**(3): p. 301-3.

42. Ey, S., Moffit, M., Kinzie, J.M., Choi, D., Girard, D.E., *"If you build it, they will come": attitudes of medical residents and fellows about seeking services in a resident wellness program.* Journal of Graduate Medical Education, 2013. **5**(3): p. 486-92.

43. Ey, S., Moffit, M., Kinzie, J.M., Brunett, P.H., *Feasibility of a Comprehensive Wellness and Suicide Prevention Program: A Decade of Caring for Physicians in Training and Practice.* Journal of graduate medical education, 2016. **8**(5): p. 747-753.

44. Feld, J., Heyse-Moore, L., *An evaluation of a support group for junior doctors working in palliative medicine.* The American journal of hospice & palliative care, 2006. **23**(4): p. 287-96.

45. Firth-Cozens, J., *Improving the health of psychiatrists.* Advances in Psychiatric Treatment, 2007. **13**(3): p. 161-168.

46. Flowers, L.K., *The missing curriculum: experience with emotional competence education and training for premedical and medical students.* Journal of the National Medical Association, 2005. **97**(9): p. 1280-7.

47. Foster, E., Biery, N., Dostal, J., Larson, D., *RAFT (Resident Assessment Facilitation Team): supporting resident well-being through an integrated advising and assessment process.* Family medicine, 2012. **44**(10): p. 731-4.

48. Burnard, P., Edwards, D., Fothergill, A., *Stress, burnout, coping and stress management in psychiatrists: findings from a systematic review.* International Journal of Social Psychiatry, 2004. **50**(1): p. 54-65.

49. Gardiner, P., Filippelli, A.C., Lebensohn, P., Bonakdar, R., *The incorporation of stress management programming into family medicine residencies-results of a national survey of residency directors: a CERA study.* Family medicine, 2015. **47**(4): p. 272-8.

50. Gardiner, M., Kearns, H., Tiggemann, M., *Effectiveness of cognitive behavioural coaching in improving the well-being and retention of rural general practitioners.* The Australian journal of rural health, 2013. **21**(3): p. 183-9.

51. Garelick, A.I., Gross, S.R., Richardson, I., von der Tann, M., Bland, J., Hale, R., *Which doctors and with what problems contact a specialist service for doctors? A cross sectional investigation.* BMC medicine, 2007. **5**: p. 26.

52. Garside, B., *Physicians mutual aid group: a response to AIDS-related burnout.* Health & social work, 1993. **18**(4): p. 259-67.

53. Gazelle, G., Liebschutz, J.M., Riess, H., *Physician burnout: coaching a way out.* Journal of general internal medicine, 2015. **30**(4): p. 508-13.

54. Geller, G., Bernhardt, B.A., Carrese, J., Rushton, C.H., Kolodner, K., *What do clinicians derive from partnering with their patients? A reliable and valid measure of "personal meaning in patient care".* Patient education and counseling, 2008. **72**(2): p. 293-300.

55. George, D.R., Dellasega, C., Whitehead, M., *Facebook stress management group for Year 1 medical students.* Medical Education, 2012. **46**(11): p. 1118.

56. Gerada, C., *For doctors with mental illness, ‘help me’ can be the hardest words*. 2018, <https://www.theguardian.com/commentisfree/2018/jun/06/doctors-mental-health-problems-taboo>: The Guardian

57. Goldhagen, B.E., Kingsolver, K., Stinnett, S.S., Rosdahl, J.A., *Stress and burnout in residents: impact of mindfulness-based resilience training.* Advances in Medical Education & Practice, 2015. **6**: p. 525-32.

58. Goodman, M.J., Schorling, J.B., *A mindfulness course decreases burnout and improves well-being among healthcare providers.* International journal of psychiatry in medicine, 2012. **43**(2): p. 119-28.

59. Graham, J., Ramirez, A.J., Field, S., Richards, M.A., *Job stress and satisfaction among clinical radiologists.* Clinical Radiology, 2000. **55**(3): p. 182-185.

60. Gregory, S.T., Menser, T., *Burnout Among Primary Care Physicians: A Test of the Areas of Worklife Model.* Journal of healthcare management / American College of Healthcare Executives, 2015. **60**(2): p. 133-48.

61. Gulen, B., Serinken, M., Eken, C., et al., *Serum S100B as a Surrogate Biomarker in the Diagnoses of Burnout and Depression in Emergency Medicine Residents.* Academic emergency medicine : official journal of the Society for Academic Emergency Medicine, 2016. **23**(7): p. 786-9.

62. Gunasingam, N., Burns, K., Edwards, J., Dinh, M., Walton, M., *Reducing stress and burnout in junior doctors: the impact of debriefing sessions.* Postgraduate medical journal, 2015. **91**(1074): p. 182-7.

63. Haizlip, J., May, N., Schorling, J., Williams, A., Plews-Ogan, M., *Perspective: the negativity bias, medical education, and the culture of academic medicine: why culture change is hard.* Academic medicine : journal of the Association of American Medical Colleges, 2012. **87**(9): p. 1205-9.

64. Hamader, G., Noehammer, E., *Prevention of anxiety, depression and burnout during medical studies and residency training (experts' opinion, medical students' and young doctors' point of view).* Noehammer, Elisabeth [Ed] (2013) Psychology of well-being: Theory, perspectives and practice (pp 33-42) xii, 229 pp Hauppauge, NY, US: Nova Science Publishers; US, 2013: p. 33-42.

65. Haramati, A., Cotton, S., Padmore, J.S., Wald, H.S., Weissinger, P.A., *Strategies to promote resilience, empathy and well-being in the health professions: Insights from the 2015 CENTILE Conference.* Medical teacher, 2017. **39**(2): p. 118-119.

66. Harrison, R., Anderson, J., Laloë, P.A., Santillo, M., Lawton, R., Wright, J., *Mentorship for newly appointed consultants: What makes it work?* Postgraduate Medical Journal, 2014. **90**(1066): p. 439-445.

67. Haward, R., Amir, Z., Borrill, C., et al., *Breast cancer teams: The impact of constitution, new cancer workload, and methods of operation on their effectiveness.* British Journal of Cancer, 2003. **89**(1): p. 15-22.

68. Hegenbarth, C., *Rescuing doctors in distress.* Canadian Medical Association Journal, 2011. **183**(3): p. E153-E154.

69. Hill, R.C., Dempster, M., Donnelly, M., McCorry, N.K., *Improving the wellbeing of staff who work in palliative care settings: A systematic review of psychosocial interventions.* Palliative Medicine, 2016. **30**(9): p. 825-833.

70. Hill, J.D., Smith, R.J.H., *Monitoring stress levels in postgraduate medical training.* The Laryngoscope, 2009. **119**(1): p. 75-8.

71. Hlubocky, F.J., Back, A.L., Shanafelt, T.D., *Addressing Burnout in Oncology: Why Cancer Care Clinicians Are At Risk, What Individuals Can Do, and How Organizations Can Respond.* American Society of Clinical Oncology educational book. American Society of Clinical Oncology. Meeting, 2016. **35**: p. 271-9.

72. Hochberg, M.S., Berman, R.S., Kalet, A.L., Zabar, S.R., Gillespie, C., Pachter, H.L., *The stress of residency: recognizing the signs of depression and suicide in you and your fellow residents.* American journal of surgery, 2013. **205**(2): p. 141-6.

73. Holoshitz, N., Wann, S., *Burnout-There's an App for That: Helping Physicians Deal With Job-Related Stress.* JAMA Cardiology, 2017. **2**(11): p. 1185-1186.

74. Horsfall, S., *Doctors who commit suicide while under GMC fitness to practice investigation.* General Medical Council, 2014.

75. Hotchkiss, N.F., *Predictors of reactivation and gender differences at a physician health program by age, marital status, primary presenting problem, referral source, and referral status.* Dissertation Abstracts International: Section B: The Sciences and Engineering, 2008. **69**(2-B): p. 1369.

76. Howlett, M., Doody, K., Murray, J., LeBlanc-Duchin, D., Fraser, J., Atkinson, P.R., *Burnout in emergency department healthcare professionals is associated with coping style: a cross-sectional survey.* Emergency medicine journal : EMJ, 2015. **32**(9): p. 722-7.

77. Ireland, M.J., Clough, B., Gill, K., Langan, F., O'Connor, A., Spencer, L., *A randomized controlled trial of mindfulness to reduce stress and burnout among intern medical practitioners.* Medical Teacher, 2017. **39**(4): p. 409-414.

78. Ro, K.E.I., Tyssen, R., Hoffart, A., Sexton, H., Aasland, O.G., Gude, T., *A three-year cohort study of the relationships between coping, job stress and burnout after a counselling intervention for help-seeking physicians.* BMC public health, 2010. **10**: p. 213.

79. Ro, K.E.I., Tyssen, R., Gude, T., Aasland, O.G., *Will sick leave after a counselling intervention prevent later burnout? A 3-year follow-up study of Norwegian doctors.* Scandinavian journal of public health, 2012. **40**(3): p. 278-85.

80. Kemper, K.J., Khirallah, M., *Acute Effects of Online Mind-Body Skills Training on Resilience, Mindfulness, and Empathy.* Journal of evidence-based complementary & alternative medicine, 2015. **20**(4): p. 247-53.

81. Kjeldmand, D., Holmstrom, I., *Balint groups as a means to increase job satisfaction and prevent burnout among general practitioners.* Annals of family medicine, 2008. **6**(2): p. 138-45.

82. Kotter, T., Pohontsch, N.J., Voltmer, E., *Stressors and starting points for health-promoting interventions in medical school from the students' perspective: a qualitative study.* Perspectives on Medical Education, 2015. **4**(3): p. 128-35.

83. Krasner, M.S., Epstein, R.M., Beckman, H., et al., *Association of an educational program in mindful communication with burnout, empathy, and attitudes among primary care physicians.* JAMA, 2009. **302**(12): p. 1284-93.

84. Kumar, S., *Burnout in psychiatrists.* World Psychiatry, 2007. **6**(3): p. 186-9.

85. Kushnir, T., Malkinson, R., Ribak, J., *Teaching stress management skills to occupational and environmental health physicians and practitioners. A graduate-level practicum.* Journal of occupational medicine. : official publication of the Industrial Medical Association, 1994. **36**(12): p. 1335-40.

86. Lapa, T.A., Carvalho, S.A., Viana, J.S., Ferreira, P.L., Pinto-Gouveia, J., *Stressors in anaesthesiology: development and validation of a new questionnaire: A cross-sectional study of Portuguese anaesthesiologists.* European Journal of Anaesthesiology, 2016. **33**(11): p. 807-815.

87. Lederer, W., Kinzl, J.F., Traweger, C., Dosch, J., Sumann, G., *Fully developed burnout and burnout risk in intensive care personnel at a university hospital.* Anaesthesia and intensive care, 2008. **36**(2): p. 208-13.

88. Lee, Y.-C., Huang, S.-C., Huang, C.-H., Wu, H.-H., *A New Approach to Identify High Burnout Medical Staffs by Kernel K-Means Cluster Analysis in a Regional Teaching Hospital in Taiwan.* Inquiry : a journal of medical care organization, provision and financing, 2016. **53**.

89. Lefebvre, D.C., *Perspective: Resident physician wellness: a new hope.* Academic medicine : journal of the Association of American Medical Colleges, 2012. **87**(5): p. 598-602.

90. Leff, V., Klement, A., Galanos, A., *A Successful Debrief Program for House Staff.* Journal Of Social Work In End-Of-Life & Palliative Care, 2017. **13**(2-3): p. 87-90.

91. Lim, R.C.H., Pinto, C., *Work stress, satisfaction and burnout in New Zealand radiologists: comparison of public hospital and private practice in New Zealand.* Journal of medical imaging and radiation oncology, 2009. **53**(2): p. 194-9.

92. Linzer, M., Poplau, S., Grossman, E., et al., *A Cluster Randomized Trial of Interventions to Improve Work Conditions and Clinician Burnout in Primary Care: Results from the Healthy Work Place (HWP) Study.* Journal of general internal medicine, 2015. **30**(8): p. 1105-11.

93. Linzer, M., Visser, M.R., Oort, F.J., et al., *Predicting and preventing physician burnout: results from the United States and the Netherlands.* The American journal of medicine, 2001. **111**(2): p. 170-5.

94. Luthar, S.S., Curlee, A., Tye, S.J., Engelman, J.C., Stonnington, C.M., *Fostering Resilience among Mothers under Stress: "Authentic Connections Groups" for Medical Professionals.* Womens Health Issues, 2017. **27**(3): p. 382-390.

95. Lyons, B., Dolezal, L., *Shame, stigma and medicine.* Medical Humanities, 2017. **43**(4): p. 208.

96. Mache, S., Vitzthum, K., Hauschild, I., Groneberg, D., *A pilot study evaluation of psychosocial competency training for junior physicians working in oncology and hematology.* Psycho-Oncology, 2017. **26**(11): p. 1894-1900.

97. Maslach, C., Leiter, M.P., *New insights into burnout and health care: Strategies for improving civility and alleviating burnout.* Med Teach, 2017. **39**(2): p. 160-163.

98. McCartney, M., *Margaret McCartney: We should stop fuelling anxiety.* BMJ, 2018. **361**.

99. McClafferty, H., Brown, O.W., *Physician health and wellness.* Pediatrics, 2014. **134**(4): p. 830-835.

100. McCray, L.W., Cronholm, P.F., Bogner, H.R., Gallo, J.J., Neill, R.A., *Resident physician burnout: is there hope?* Family medicine, 2008. **40**(9): p. 626-32.

101. McCue, J.D., Sachs, C.L., *A stress management workshop improves residents' coping skills.* Archives of internal medicine, 1991. **151**(11): p. 2273-7.

102. McKenna, K.M., Hashimoto, D.A., Maguire, M.S., Bynum, W.E.t., *The Missing Link: Connection Is the Key to Resilience in Medical Education.* Academic medicine : journal of the Association of American Medical Colleges, 2016. **91**(9): p. 1197-9.

103. McKevitt, C., Morgan, M., *Illness doesn't belong to us.* Journal of the Royal Society of Medicine, 1997. **90**(9): p. 491-495.

104. McKinley, T.F., Boland, K.A., Mahan, J.D., *Burnout and interventions in pediatric residency: A literature review.* Burnout Research, 2017. **6**: p. 9-17.

105. McManus, I.C., Jonvik, H., Richards, P., Paice, E., *Vocation and avocation: leisure activities correlate with professional engagement, but not burnout, in a cross-sectional survey of UK doctors.* BMC medicine, 2011. **9**: p. 100.

106. McNeill, K.G., Kerr, A., Mavor, K.I., *Identity and norms: the role of group membership in medical student wellbeing.* Perspectives on Medical Education, 2014. **3**(2): p. 101-12.

107. Mechaber, H.F., Levine, R.B., Manwell, L.B., et al., *Part-time physicians...prevalent, connected, and satisfied.* Journal of general internal medicine, 2008. **23**(3): p. 300-3.

108. Meerten, M., Rost, F., Bland, J., Garelick, A.I., *Self-referrals to a doctors' mental health service over 10 years.* Occupational medicine (Oxford, England), 2014. **64**(3): p. 172-6.

109. Mehta, D.H., Perez, G.K., Traeger, L., et al., *Building Resiliency in a Palliative Care Team: A Pilot Study.* Journal of pain and symptom management, 2016. **51**(3): p. 604-8.

110. Milstein, J.M., Raingruber, B.J., Bennett, S.H., Kon, A.A., Winn, C.A., Paterniti, D.A., *Burnout assessment in house officers: evaluation of an intervention to reduce stress.* Medical teacher, 2009. **31**(4): p. 338-41.

111. Montgomery, A., Panagopoulou, E., Kehoe, I., Valkanos, E., *Connecting organisational culture and quality of care in the hospital: is job burnout the missing link?* Journal of Health Organization and Management, 2011. **25**(1): p. 108-23.

112. Moutier, C., Norcross, W., Jong, P., et al., *The suicide prevention and depression awareness program at the University of California, San Diego School of Medicine.* Academic medicine : journal of the Association of American Medical Colleges, 2012. **87**(3): p. 320-6.

113. Murdoch, J.M., Eagles, J.M., *'Stress-busting' groups for consultant psychiatrists.* Psychiatric Bulletin, 2007. **31**(4): p. 128-131.

114. Myszkowski, N., Villoing, B., Zenasni, F., Jaury, P., Boujut, E., *Monitoring stress among internal medicine residents: an experience-driven, practical and short measure.* Psychology, Health & Medicine, 2017. **22**(6): p. 719-726.

115. NHS England, *NHS Staff Health and Wellbeing: CQUIN Supplementary Guidance; 2016*. 2016.

116. Nielsen, H.G., Tulinius, C., *Preventing burnout among general practitioners: is there a possible route?* Education for primary care : an official publication of the Association of Course Organisers, National Association of GP Tutors, World Organisation of Family Doctors, 2009. **20**(5): p. 353-9.

117. Nomura, O., Mishina, H., Kobayashi, Y., Ishiguro, A., Sakai, H., Kato, H., *Limitation of duty hour regulations for pediatric resident wellness: A mixed methods study in Japan.* Medicine, 2016. **95**(37): p. e4867.

118. Oczkowski, S., *Virtuous laughter: we should teach medical learners the art of humor.* Critical care (London, England), 2015. **19**: p. 222.

119. Oman, D., Hedberg, J., Thoresen, C.E., *Passage meditation reduces perceived stress in health professionals: a randomized, controlled trial.* Journal of consulting and clinical psychology, 2006. **74**(4): p. 714-9.

120. Paice, E., Aitken, M., Houghton, A., Firth-Cozens, J., *Bullying among doctors in training: Cross sectional questionnaire survey.* BMJ: British Medical Journal, 2004. **329**(7467): p. 658-659.

121. Paice, E., Rutter, H., Wetherell, M., Winder, B., McManus, I.C., *Stressful incidents, stress and coping strategies in the pre-registration house officer year.* Medical Education, 2002. **36**(1): p. 56-65.

122. Panagioti, M., Panagopoulou, E., Bower, P., et al., *Controlled interventions to reduce burnout in physicians: A systematic review and meta-analysis.* JAMA Internal Medicine, 2017. **177**(2): p. 195-205.

123. Penfold, R., *Why junior doctors need more autonomy.* BMJ, 2018. **363**.

124. Pereira, M.A.D., Barbosa, M.A., de Rezende, J.C., Damiano, R.F., *Medical student stress: an elective course as a possibility of help.* BMC research notes, 2015. **8**: p. 430.

125. Prins, J.T., Hoekstra-Weebers, J.E.H.M., Gazendam-Donofrio, S.M., et al., *The role of social support in burnout among Dutch medical residents.* Psychology, Health & Medicine, 2007. **12**(1): p. 1-6.

126. Rabin, S., Matalon, A., Maoz, B., Shiber, A., *Keeping doctors healthy: A salutogenic perspective.* Families, Systems, & Health, 2005. **23**(1): p. 94-102.

127. Raj, K.S., *Well-Being in Residency: A Systematic Review.* Journal of graduate medical education, 2016. **8**(5): p. 674-684.

128. Ramirez, A.J., Graham, J., Richards, M.A., Cull, A., *Mental health of hospital consultants: the effects of stress and satisfaction at work.* Lancet, 1996. **347**: p. 724-728.

129. Ro, K.I., Veggeland, F., Aasland, O.G., *Peer counselling for doctors in Norway: A qualitative study of the relationship between support and surveillance.* Social Science & Medicine, 2016. **162**: p. 193-200.

130. Regehr, C., Glancy, D., Pitts, A., LeBlanc, V.R., *Interventions to reduce the consequences of stress in physicians: a review and meta-analysis.* The Journal of nervous and mental disease, 2014. **202**(5): p. 353-9.

131. Riley, R., Spiers, J., Buszewicz, M., Taylor, A.K., Thornton, G., Chew-Graham, C.A., *What are the sources of stress and distress for general practitioners working in England? A qualitative study.* BMJ Open, 2018a. **8**(1).

132. Riley, R., Spiers, J., Chew-Graham, C.A., Taylor, A.K., Thornton, G.A., Buszewicz, M., *‘Treading water but drowning slowly’: what are GPs’ experiences of living and working with mental illness and distress in England? A qualitative study.* BMJ open, 2018b. **8**(5): p. e018620.

133. Ringrose, R., Houterman, S., Koops, W., Oei, G., *Burnout in medical residents: A questionnaire and interview study.* Psychology, Health & Medicine, 2009. **14**(4): p. 476-486.

134. Ripp, J.A., Fallar, R., Korenstein, D., *A Randomized Controlled Trial to Decrease Job Burnout in First-Year Internal Medicine Residents Using a Facilitated Discussion Group Intervention.* Journal of graduate medical education, 2016. **8**(2): p. 256-9.

135. Ripp, J.A., Privitera, M.R., West, C.P., et al., *Well-Being in Graduate Medical Education: A Call for Action.* Academic medicine : journal of the Association of American Medical Colleges, 2017. **92**(7): p. 914-917.

136. Ripp, J.A., Bellini, L., Fallar, R., Bazari, H., Katz, J.T., Korenstein, D., *The impact of duty hours restrictions on job burnout in internal medicine residents: a three-institution comparison study.* Academic medicine : journal of the Association of American Medical Colleges, 2015. **90**(4): p. 494-9.

137. Rippstein-Leuenberger, K., Mauthner, O., Bryan Sexton, J., Schwendimann, R., *A qualitative analysis of the Three Good Things intervention in healthcare workers.* BMJ Open, 2017. **7**(5): p. e015826.

138. Ro, K.E.I., Gude, T., Tyssen, R., Aasland, O.G., *Counselling for burnout in Norwegian doctors: one year cohort study.* BMJ (Clinical research ed.), 2008. **337**: p. a2004.

139. Roberts, G., Moore, B., Coles, C., *Mentoring for newly appointed consultant psychiatrists.* Psychiatric Bulletin, 2002. **26**(3): p. 106-109.

140. Robertson, I., Cooper, C., *The Boorman Report on the Health and Well-Being of NHS Staff: Practical advice for implementing its recommendations.* 2010.

141. Rohland, B.M., Kruse, G.R., Rohrer, J.E., *Validation of a single-item measure of burnout against the Maslach Burnout Inventory among physicians.* Stress and Health: Journal of the International Society for the Investigation of Stress, 2004. **20**(2): p. 75-79.

142. Rothenberger, D.A., *Physician Burnout and Well-Being: A Systematic Review and Framework for Action.* Diseases of the colon and rectum, 2017. **60**(6): p. 567-576.

143. Runyan, C., Savageau, J.A., Potts, S., Weinreb, L., *Impact of a family medicine resident wellness curriculum: a feasibility study.* Medical education online, 2016. **21**(1): p. 30648.

144. Sallon, S., Katz-Eisner, D., Yaffe, H., Bdolah-Abram, T., *Caring for the Caregivers: Results of an Extended, Five-component Stress-reduction Intervention for Hospital Staff.* Behavioral Medicine, 2017. **43**(1): p. 47-60.

145. Sanchez, L.T., Candilis, P.J., Arnstein, F., et al., *Effectiveness of a Unique Support Group for Physicians in a Physician Health Program.* Journal of psychiatric practice, 2016. **22**(1): p. 56-63.

146. Schapira, L., Meisel, J.L., Srivastava, R., *For Our Patients, for Ourselves: The Value of Personal Reflection in Oncology.* American Society of Clinical Oncology Educational Book, 2017. **37**: p. 765-770.

147. Schattner, A., *Residents' responsibilities: Adopting a wider view.* Medical Teacher, 2017. **39**(12): p. 1286-1289.

148. Schmitz, G.R., Clark, M., Heron, S., et al., *Strategies for coping with stress in emergency medicine: Early education is vital.* Journal of Emergencies Trauma & Shock, 2012. **5**(1): p. 64-9.

149. Schneider, S., Kingsolver, K., Rosdahl, J., *Physician coaching to enhance well-being: a qualitative analysis of a pilot intervention.* Explore (New York, N.Y.), 2014. **10**(6): p. 372-9.

150. Scholz, M., Neumann, C., Wild, K., et al., *Teaching to Relax: Development of a Program to Potentiate Stress-Results of a Feasibility Study with Medical Undergraduate Students.* Applied Psychophysiology & Biofeedback, 2016. **41**(3): p. 275-81.

151. Seoane, L., Tompkins, L.M., De Conciliis, A., Boysen, P.G., 2nd, *Virtues Education in Medical School: The Foundation for Professional Formation.* Ochsner Journal, 2016. **16**(1): p. 50-5.

152. Shanafelt, T.D., Oreskovich, M.R., Dyrbye, L.N., et al., *Avoiding burnout: the personal health habits and wellness practices of US surgeons.* Annals of surgery, 2012. **255**(4): p. 625-33.

153. Shanafelt, T.D., Kaups, K.L., Nelson, H., et al., *An interactive individualized intervention to promote behavioral change to increase personal well-being in US surgeons.* Annals of surgery, 2014. **259**(1): p. 82-8.

154. Shapiro, J., Astin, J., Shapiro, S.L., Robitshek, D., Shapiro, D.H., *Coping with loss of control in the practice of medicine.* Families, systems & health : the journal of collaborative family healthcare, 2011. **29**(1): p. 15-28.

155. Shapiro, J., Galowitz, P., *Peer Support for Clinicians: A Programmatic Approach.* Academic medicine : journal of the Association of American Medical Colleges, 2016. **91**(9): p. 1200-4.

156. Sharifi, P.S., *Fostering self-care and mindfulness in mental health care providers.* Dissertation Abstracts International: Section B: The Sciences and Engineering, 2013. **73**(12-B(E)): p. No Pagination Specified.

157. Shiralkar, M.T., Harris, T.B., Eddins-Folensbee, F.F., Coverdale, J.H., *A systematic review of stress-management programs for medical students.* Academic psychiatry : the journal of the American Association of Directors of Psychiatric Residency Training and the Association for Academic Psychiatry, 2013. **37**(3): p. 158-64.

158. Siedsma, M., Emlet, L., *Physician burnout: can we make a difference together?* Critical care (London, England), 2015. **19**: p. 273.

159. Sigsbee, B., Bernat, J.L., *Physician burnout: A neurologic crisis.* Neurology, 2014. **83**(24): p. 2302-6.

160. Slavin, S.J., Chibnall, J.T., *Finding the Why, Changing the How: Improving the Mental Health of Medical Students, Residents, and Physicians.* Academic medicine : journal of the Association of American Medical Colleges, 2016. **91**(9): p. 1194-6.

161. Slavin, S.J., Hatchett, L., Chibnall, J.T., Schindler, D., Fendell, G., *Helping medical students and residents flourish: A path to transform medical education.* Academic Medicine, 2011. **86**(11): p. e15.

162. Smith, S., *Breaking down the barriers of stigma: Understanding and fostering help-seeking behaviors in medical students.* Dissertation Abstracts International Section A: Humanities and Social Sciences, 2017. **77**(9-A(E)): p. No Pagination Specified.

163. Squiers, J.J., Lobdell, K.W., Fann, J.I., DiMaio, J.M., *Physician Burnout: Are We Treating the Symptoms Instead of the Disease?* The Annals of thoracic surgery, 2017. **104**(4): p. 1117-1122.

164. Talisman, N., Harazduk, N., Rush, C., Graves, K., Haramati, A., *The impact of mind-body medicine facilitation on affirming and enhancing professional identity in health care professions faculty.* Academic medicine : journal of the Association of American Medical Colleges, 2015. **90**(6): p. 780-4.

165. Tucker, T., Bouvette, M., Daly, S., Grassau, P., *Finding the sweet spot: Developing, implementing and evaluating a burn out and compassion fatigue intervention for third year medical trainees.* Evaluation & Program Planning, 2017. **65**: p. 106-112.

166. van Vliet, M., Jong, M., Jong, M.C., *Long-term benefits by a mind-body medicine skills course on perceived stress and empathy among medical and nursing students.* Medical Teacher, 2017. **39**(7): p. 710-719.

167. Verweij, H., Waumans, R.C., Smeijers, D., et al., *Mindfulness-based stress reduction for GPs: results of a controlled mixed methods pilot study in Dutch primary care.* The British journal of general practice : the journal of the Royal College of General Practitioners, 2016. **66**(643): p. e99-105.

168. Waddimba, A.C., Scribani, M., Nieves, M.A., Krupa, N., May, J.J., Jenkins, P., *Validation of Single-Item Screening Measures for Provider Burnout in a Rural Health Care Network.* Evaluation & the Health Professions, 2016. **39**(2): p. 215-25.

169. Wald, H.S., Haramati, A., Bachner, Y.G., Urkin, J., *Promoting resiliency for interprofessional faculty and senior medical students: Outcomes of a workshop using mind-body medicine and interactive reflective writing.* Medical teacher, 2016. **38**(5): p. 525-8.

170. Warde, C.M., Vermillion, M., Uijtdehaage, S., *A medical student leadership course led to teamwork, advocacy, and mindfulness.* Family medicine, 2014. **46**(6): p. 459-62.

171. West, C.P., Dyrbye, L.N., Rabatin, J.T., et al., *Intervention to promote physician well-being, job satisfaction, and professionalism: a randomized clinical trial.* JAMA internal medicine, 2014. **174**(4): p. 527-33.

172. West, C.P., Dyrbye, L.N., Erwin, P.J., Shanafelt, T.D., *Interventions to prevent and reduce physician burnout: a systematic review and meta-analysis.* Lancet (London, England), 2016. **388**(10057): p. 2272-2281.

173. Wild, K., Scholz, M., Ropohl, A., Brauer, L., Paulsen, F., Burger, P.H.M., *Strategies against burnout and anxiety in medical education--implementation and evaluation of a new course on relaxation techniques (Relacs) for medical students.* PloS one, 2014. **9**(12): p. e114967.

174. Wilkie, G., Raffaelli, D., *In at the deep end: Making the transition from SpR to consultant.* Advances in Psychiatric Treatment, 2005. **11**(2): p. 107-114.

175. Williams, S., Michie, S., Pattani, S., *Improving the health of the NHS workforce: Report of the partnership on the health of the NHS workforce*. 1998: Nuffield Trust.

176. Winefield, H., Farmer, E., Denson, L., *Work stress management for women general practitioners: An evaluation.* Psychology, Health & Medicine, 1998. **3**(2): p. 163-170.

177. Wolf, T., *Stress, coping and health: Enhancing well-being during medical school.* Medical Education, 1994. **28**(1): p. 8-17.

178. Zhang, H., Isaac, A., Wright, E.D., Alrajhi, Y., Seikaly, H., *Formal mentorship in a surgical residency training program: a prospective interventional study.* Journal of Otolaryngology: Head and Neck Surgery, 2017. **46**(1): p. 13.

179. Zwack, J., Schweitzer, J., *If every fifth physician is affected by burnout, what about the other four? Resilience strategies of experienced physicians.* Academic medicine : journal of the Association of American Medical Colleges, 2013. **88**(3): p. 382-9.
