# Supplementary material for: Optimising strategies to address mental ill-health in doctors and medical students: ‘Care Under Pressure’ realist review and implementation guidance
Source: BMC Med. 2020 Apr 8;18:76. doi: 10.1186/s12916-020-01532-x (PMC7106831; doi:10.1186/s12916-020-01532-x)
Supplement: Supplementary file 4 — Additional file 4. Examples of the citations that provide evidence for each CMOc. [file 12916_2020_1532_MOESM4_ESM.docx]

## **CMOc1: Underdeveloped workforce planning**

In a workplace in which basic support structures to enable doctors to do their job are not in place (C), doctors may feel that they must make up for the deficiencies of the organisation for patients and colleagues (M). This may contribute to a toxic working culture in which overwork and its negative consequences are normalised (O).

## **Relevant extracts from papers included in the review**

There were inconsistencies in the range of services offered by different NHS Trusts, and many services suffered from staff shortages and inadequate resourcing, with funding often historically based rather than related to current needs [1] ***Policy document UK, NHS workforce, 2009***

Trainees [..] reported [...] seniors in a working culture ‘institutionally opposed’ to hearing complaints; doctors[…] emotionally blackmailed or bullied into reporting only timetabled hours, […] individuals made to feel that working excessive hours was a personal failure of time management rather than the consequence of a systemic mismatch of workload.[2]
***Research, UK, doctors, trainee, consultant, 2014***

Physicians, it seems, have become quite skilled at sacrificing personal and family time in service to patients and the increasing demands of practice. [3]
***Commentary, USA, Primary care, trainees, consultants, medical students, 2015***

If burnout is a problem of whole health care systems, it is less likely to be effectively minimized by solely intervening at the individual level. It requires an organization-embedded approach.[4]
***Research, UK, physicians, consultants, trainees, 2017***

## **CMOc2: Normalisation of high workload**

When high workload and its negative consequences (e.g. distress, burnout) are normalised (C), overworked or sick doctors may feel they are letting down their colleagues and patients (M). This can contribute to presenteeism (O) and associated negative consequences on mental health (O1) and workforce retention (O2).

## **Relevant extracts from papers included in the review**

Importantly, presenteeism is unlikely to decrease if individuals are operating in environments where working through illness is viewed as ‘normal’ or, at worst, ‘necessary’ behaviour [5]
***Research, New Zealand, doctors and dentists, consultant, 2017***

All doctors referred to the 'burden' which their absence placed on colleagues, who would be expected to assume their duties [6]
***Research, UK, doctors, trainees, consultants, medical students, 1997***

## **CMOc3: Loss of autonomy**

When doctors experience lack of autonomy over their work (C1), and some aspects of their work as less meaningful (C2) they may feel dissatisfied with their job (e.g. because they are unable to do the job they were trained for) (M). This can make doctors more vulnerable to stress and mental ill-health, irrespective of workload (O)

## **Relevant extracts from papers included in the review**

Although most efforts at preventing physician burnout are focused on improving individual physician resilience, health care organizations are failing to change the system that is increasingly asking doctors to perform tasks, largely administrative in nature, for which they have no passion.[7]
***Research, USA, physicians, trainees, consultants, 2017***

‘You spent more time ticking boxes than you did talking to the patients sometimes […] that put more stress on me and I felt it affected my rapport with the patients.’[8]
***Research, UK, GPs, consultants, 2016***

This study confirms that autonomy and feeling well managed and resourced make a substantial contribution to overall job satisfaction. […], the transfer of various responsibilities from clinicians to managers may undermine professional morale, as may the practice of establishing standards against which doctors’ performance is judged, without their involvement[9]
***Research, UK, gastroenterologists, surgeons, radiologists oncologists, consultants, trainees, 1996***

**CMOc4: Stigma towards vulnerability**

In a professional culture where mental ill-health and vulnerability may be seen as unprofessional (C) doctors (and medical students) may feel ashamed (M1) or afraid (M2) of not living up to their professional identity if they experience mental ill-health (or other difficulties at work). This can lead doctors (and medical students) to adopt strategies which involve hiding their difficulties from themselves and colleagues (O).

**Relevant extracts from papers included in the review**

“Illness doesn't belong to us. It belongs to them, the patients. Doctors need to be taught to be ill. We need permission to be ill and to acknowledge that we are not superhuman”.[6]

***Research, UK, doctors, trainee, consultant, medical student, 1997***

Perhaps symptomatic of the stigma attached to mental illness and the culture of invulnerability within medicine, some GP participants described how they felt ashamed, embarrassed, humiliated and a sense of having failed, due to their perceived lack of resilience and inability to cope.[10]

***Research, UK, GPs, trainees, consultants, 2018***

there is a cultural expectation within medicine that doctors do not expect themselves or their colleagues to be sick, with only one-third of junior UK doctors registered with a general practitioner […],worries about conﬁdentiality can lead doctors to take responsibility for their own care.[11] ***Research, UK/Greece, hospital staff, trainees, consultants, 2011***

Medical training emphasizes perfectionism, denial of personal vulnerability, and delayed gratification. Traits such as compulsiveness, guilt, and self-denial may facilitate success in medical education and training; however, in a long-term career, these same traits can fuel feelings of inadequacy. […] these factors contribute to burnout [12]
***Commentary, USA, physicians, trainees, consultants, 2015***

Medical students often avoid seeking help because they believe it might affect their future career plans or fear that they will be seen as “weak” by others [13]
***Research, USA, medical students, 2013***

## **CMOc5: Hiding vulnerability**

Where there is mental health support available for doctors (C1), doctors, who understand the system and that confidentiality is difficult to achieve (C2), may fear that seeking support could jeopardise their career (M) and so they may hide their distress rather than seek support (O).

## **Relevant extracts from papers included in the review**

…this study reported that only 1 in 4 of these at-risk surgeons sought professional help. Those who did not seek help said their reticence to do so (among other reasons) stemmed from the requirement of some state-licensing boards to report their personal psychiatric/psychological care.[14]
***Intervention, USA, surgeons, trainees, consultants, 2013***

evidence suggests that providers experiencing suicidal thoughts may be less likely to seek mental health care because of concerns about their medical licensure.34 (The same providers are, however, more likely to self-prescribe antidepressants.34)[15]
***Research, USA, medical trainees, 2016***

[doctors] do not ask for treatment early, if at all. The system […] may militate against their being able to do this easily. There are too many worries about confidentiality and potential impact on career and colleagues for many doctors to do what they would tell their patients to do.[16]
***Policy, UK, doctors, trainees, consultants, medical students, 2008***

## **CMOc6: Isolation**

When doctors work in physical and emotional isolation (C) they are likely to feel less supported by their colleagues and/or their employing organisation (M1), and/or mistrust of these groups (M2). This can make doctors more vulnerable to work-related pressure and mental ill-health (O).

## **Relevant extracts from papers included in the review**

Physicians are increasingly isolated—physically because they work in diverse settings or emotionally because there is little time for learning about each other’s stresses, discussing challenging professional situations, and developing personal connections. This sense of isolation may be exacerbated as […]electronic documentation is rapidly replacing collegial face-to-face communication.[…].[17]
***Commentary, USA, doctors and other HCPs, 2013***

disconnection is the single greatest threat to our efforts to foster resilience and to promote
 wellness in medical education.[18]
 ***Commentary, USA, medical students, 2016***

Medicine is a collective venture (Kivimaki et al., 2001) and when working relationships become impaired the ability to manage one’s tasks as a doctor in a complex working environment becomes almost impossible.[19]
***Intervention, UK, doctors, consultants, trainees, 2016***

## **CMOc7: Positive and meaningful workplace relations**

Positive and meaningful workplace relations (C) can foster a sense of belonging between colleagues and towards the medical profession (M). This can lead to an increased capacity to work under pressure (O)

## **Relevant extracts from papers included in the review**

In medicine, group membership, or “being part of the club,” can serve as a much-needed safety net when adversity overwhelms a learner’s individual resilience. […] [18]
***Commentary, USA, medical students, 2016***

According to qualitative studies, being part of a Balint group helped GPs recognize their professional identity and rediscover the joy of being a physician […] participation in a Balint group reduced burnout levels and sensitivity to stress among GPs [20]
***Intervention, Israel, oncologists, trainees, consultants, 2012***

Debrieﬁng programmes are low cost, easily coordinated and potentially have the capacity to offer institutional support throughout the course of an internship. The availability of personnel trained in debrieﬁng would be a valuable asset to any unit supervising training junior doctors[21] ***Intervention, Australia, doctors, trainees, 2015***

Efforts to improve collegiality can provide better resident and staff well-being and also improve health care provider outcomes [22]
***Research, USA, paediatric residents, 2017***

Success of the palliative care service is contingent on the cohesion and wellbeing of the team members, especially as caseloads and job demands have increased [23]
***Intervention, USA, palliative care team, trainees, consultants, 2016***

Medical residency is undoubtedly stressful, and it has been compared to surviving a dysfunctional family. Negative relational experiences have been shown to affect both current learning and future patient care [24]
***Intervention, USA, doctors, trainees, consultants, 2012***

## **CMOc8: Functional working groups**

Working in functional groups (C) can make doctors feel more supported (M1) and more at ease with vulnerability (M2). This can normalise vulnerability (O1) and reduce the stigma around mental ill-health (O2)

## **Relevant extracts from papers included in the review**

Once individuals felt safe in the group, they would share more personal matters such as fear of overload when faced with a run of deaths or profound feelings of loss at the death of a patient they knew well and sadness for the family’s grief. [25]
***Intervention, UK, palliative care, trainees, consultants, 2006***

in a Balint group, the doctor can learn to recognise personal reactions, develop an awareness of the early signals of distress, and internalise what is learnt from the interaction with other participants. […]debrief, ‘normalise’ emotional reactions, reduce stress by sharing experiences, reinforce the value of their work, and reformulate boundaries[26]
***Commentary , Australia, GP, consultants, 2005***

Team working was beneficial to the mental health of its members, allowing problems and pressures to be shared, and individuals to be supported by colleagues [27]
***Research, UK, cancer teams, consultants, trainees, 2003***

“Seeing my own struggles/thoughts amongst the topics discussed – makes you feel less alone”[28]
***Intervention, Canada, medical trainees, 2017***

## **CMOc9: Balancing quality and quantity of time at work**

When doctors (for different reasons) have less connectedness and meaning at work (C), they may feel they can only find fulfilment outside work (M2), making it less likely that their condition will improve (O).

## **Relevant extracts from papers included in the review**

we should prioritize connecting with one another and with our patients to build a sense of belonging within our teams, programs, hospitals, and profession. We should focus less on the competition between work and life and more on making our work a functional and enjoyable part of our lives [18]
**Commentary, USA, medical students, 2016**

Although receiving unstructured protected time offered some benefits by itself, the advantages of the small-group curriculum were greater and persisted after the intervention concluded, particularly for meaning and the closely associated interpersonal aspects of burnout [29]
***Intervention, USA, physicians, trainees, consultants, 2014***

despite good intentions, duty hours limitations have produced little concrete benefit and have created many unintended negative consequences. Like many things in medicine, duty hours must be carefully titrated to strike a balance between resident wellness, patient safety, and quality education.[30]
***Commentary, Canada, physicians, trainees, consultants, 2012***

A straightforward practical implication is that to prevent burnout, GPs should be provided with the opportunity to restore the balance in the doctor- patient relationship..[31]
***Research, Netherlands, GPs, consultant, trainees, 2000***

## **CMOc10: Limits of groups**

Sick doctors (and medical students) with particularly delicate circumstances (C) may not feel safe to share their problems (M1) and /or may not identify with the other group members (M2). This can result in a dysfunctional group (O1) and intensification of mental ill-health in doctors (O2).

## **Relevant extracts from papers included in the review**

there are situations where a particular clinician has been at the “sharp end of an error,” and such a circumstance is not conducive to group peer support. […]. [32]
***Commentary, USA, doctors, trainees, consultants, 2016***

“I felt that my feelings were somewhat minimized. Because I was not yet having severe behavioral problems? Because I was still functional, had a good job and got along great with my staff? Experiencing death and grief—heavy loads of responsibility were not enough”.[33]
***Research, USA, physicians, trainees, consultants***

the experts interviewed pointed out the importance of intervention options at the first signs of psychological problems to avoid stigmatization and sanctions, to provide a quick anonymous psychological counselling outside of the clinic as well as to improve social acceptance of psychosocial problems[34]
***Research, Austria, medical students, occupational health and psychology, 2013***

Focus group evaluation demonstrated that students greatly valued the Facebook group, particularly the YouTube testimonials containing advice from older students.[...] The Facebook platform prompted peer-to-peer discussions about stress and engendered friendships amongst students, while also offering the beneﬁt of anonymity.[35]
***Intervention, USA, medical students, 2012***

## **CMOc11: ‘Organic’ spaces to connect**

If there are protected times and psychologically safe spaces for students/doctors to congregate within the confines of the work environment (C), students/doctors are likely to bond over whatever is most important to them at that time (M). This may improve connectedness (O)

**Relevant extracts from papers included in the review**

Medical schools often facilitate group identiﬁcation of their students by setting up activities in which students cooperate to work towards a common goal. However, the competitive nature of medical school may undermine a shared group identity […]. Strategies such as utilising a pass/ fail[…] may reduce this competitive atmosphere [36]
***Research, Australia, medical students, 2014***

Sharing a laugh is a simple pleasure and a shared experience which brings people together [4]. In a place where life and death hang in the balance, it is easy to become self-important, arrogant, or closed-minded. By poking fun at ourselves, humor can keep us humble, and keep us together [37]
***Commentary, Canada, physicians, trainees, medical students, 2006***

Writing about our experiences as oncologists can help us understand them with greater clarity. […] Reading and writing narratives in oncology may strengthen us emotionally, allowing us to be more fully present for our patients and our loved ones.[38]
***Commentary, Australia, oncologists, trainees, consultants, 2017***

## **CMOc12: Recognising both positive and negative performance**

Where supervision and feedback recognise both positive and negative performance and promote doctors’ (and students’) learning from both of these (C), doctors (and students) may feel more fairly treated (M1) and more inclined to value their colleagues and employing organisation (M2), potentially leading to more connectedness and engagement at work (O1), and a more supportive work culture (O2).

**CMOc13: Balancing prevention of mental ill-health with promotion of wellbeing**

In a work environment that actively demonstrates the importance of the balance between health and wellbeing with fighting stress and mental ill-health (C), doctors (and students) are more likely to feel that caring about their own wellbeing is legitimate (M1) and less afraid to acknowledge vulnerability (M2). This can contribute to a de-stigmatisation of mental ill-health and vulnerability (O).

## **Relevant extracts from included studies**

We must continue to improve our care of patients through evaluation of error and flawed judgment. But […] we can balance their effects by making a conscious effort also to notice the positive. Following the lead of appreciative inquiry, we can recognize successes and seek to learn how they are achieved.[39]
***Commentary, USA, consultants, trainees, 2012***

The term "mental health" has come to imply mental illness, not health, and the study of pathology has far outstripped any exploration of disease prevention and health promotion in psychiatry.[40]
***Research, USA/UK, medical students, 2005***

We recommend the following changes to the medical education system to promote students’ well-being and flourishing: […]

• Encourage and celebrate achievement.[…]

• Promote positive emotions [41]
***Commentary, USA, medical students, trainees, 2011***

We must allow doctors to become patients without the fear of sanctions or blame, and aﬀord them the same compassion as they are expected to give to their own patients.[42]
***Commentary, UK, consultants, trainees, 2018***

## **CMOc14: Acknowledging the positive and negative aspects of the profession**

Where both the positive and negative aspects of a medical career are recognised (C), doctors (and medical students) may feel less inadequate and helpless when they or their colleagues experience stress or mental ill-health (M). This may lead to increased capacity to deal with work pressure (O1) and to recognition and acceptance of vulnerability (O2).

## **Relevant extracts from included studies**

From medical school onwards, doctors need to be enabled to understand the nature of the stress that a career in medicine can bring, and the possible impact on their health, their relationships and their work.[…] [and] acquire skills that help deal with stressors without resorting to counterproductive ways of coping [43]

***Intervention, USA, medical students, 2013***

doctors […] find that their training has not prepared them for the demands of a health service that is becoming increasingly complex and less cohesive with [..] less autonomy and increased accountability.[…] The answer is not to be found within individuals but between individuals and the culture they find themselves in [44]
***Intervention, UK, doctors, trainees, consultants, 2016***

Educating medical students about the complexity and challenges of medicine, emphasizing the normality of feelings of vulnerability and stress, the acceptability of acknowledging difficulty as the first step in managing it and that seeking support is adaptive rather than a sign of weakness.[45]
***Research, UK, doctors, trainees, consultants, 2014***

It is advantageous to obtain a clear understanding and have realistic expectations about what it takes to become a doctor and the road that has to be travelled prior even to entering medical school [46]
***Research, USA, medical students, 1994***

realistic expectations of a modern career in medicine underpin contentedness in the medical workplace. Medical schools must work closely with postgraduate deans responsible for junior doctors to ensure that undergraduate education encompasses [..] also the appropriate processes to prepare doctors for the rapidly changing environment of medical practice.[47]
***Commentary, UK, medical students, 2005***

A culture in which trainees and faculty speak openly about the challenges of training and its anticipated impact will serve to promote wellness and destigmatize stress-related concerns.[48]
***Commentary, USA, medical students, 2017***

## **CMOc15: Timely support**

Timely support when doctors (and students) are particularly vulnerable (e.g. after a suicide attempt, death of a colleague, addiction) (C) may represent their only source of hope (M) and reduce the intensity of mental ill-health and its related outcomes, including suicide (O).

**Relevant extracts from included studies**

Approximately 20% of survivors of unsuccessful suicide attempts will attempt harm within a year and as a group they are twice as likely to succeed in committing suicide compared with those who have never attempted to take their own life.[49]
***Policy, UK, doctors under investigations, 2014***

Strategies to address substance abuse problems include encouraging confidential self-reporting, increasing access to treatment programs and local resources, and allowing protected feedback from co-workers to help identify problems early on.[50]
***Commentary, USA, emergency medicine, trainees, consultants, 2012***

During orientation, trainees are encouraged by RFWP *(Resident and Faculty Wellness Program)* staff and program leadership to not wait for a crisis to access counseling.[51]
***Intervention, USA, medical trainees and faculty, 2016***

provide extra support for all doctors during life events, complaints and disciplinary actions, and for psychiatrists when a patient dies by suicide – all factors known to precede the onset of depression in doctors […]the inadequacy of the help provided for mental health problems may contribute to tragic consequences [52]

***Research, UK, doctors sand psychiatrists, consultants, trainees, 2007***

## **CMOc16: Endorsement**

Doctors are less likely to engage with an intervention (O) if it is not endorsed by the employing organisation and senior leadership (C) because they may then lack trust in it (M1) and may also feel frustrated (M2) if they cannot access it due to work constraints.

## **Relevant extracts from papers included in the review**

These explanations are reflected by the respondents’ suggestions about the need for the medical profession to acknowledge that physicians can have problems and for the QPHP (Quebec Physician Health Program) services to be better known by the profession.[53]

***Research, Canada, physicians, trainees, consultants, 2010***

Programs that aim to improve resident wellness can only be effective if residents’ time is truly protected and the curriculum is not just another time demand[54]
***Research, USA, doctors, trainees, consultants, 2018***

Chief and senior residents became a significant source of referrals to the RWP[ residents wellness programme] with residents reporting, ‘‘If my chief said it helped her and urged me to come, I decided it must be okay.’’[55]
***Research, USA, doctors, trainees, 2013***

The dean himself sent a strong, consistent message to medical students, residents, fellows, and faculty emphasizing that no stigma should be attached to mental illness and encouraging everyone, sick or well, to participate in the program.[56]
***Research, USA, medical students, trainees, consultants, 2012***

The drive to deliver an effective staff health and well-being service requires board commitment, clearly identiﬁed top management leadership and staff engagement. Without this, initiatives, however well intentioned, are likely to be less than fully effective.[57]
***Policy, UK, NHS workforce, 2009***

## **CMOc17: Expertise**

If those delivering interventions do not have specific training to address the needs of sick doctors (C), the recipients may be less likely to trust the intervention (M) and the intervention may be ineffective (O1) and / or harmful (O2) or not accessed at all (O3).

## **Relevant extracts from papers included in the review**

Employing coaches who understand the culture and system of medicine is one important step towards supporting physicians in a process that prioritizes self-care [58]
***Research, USA, physicians, trainees, consultants, 2014***

Attention not only should be given to the medical residents, but also to their supervisors in developing supportive skills as well as improving their present relationship with residents.[59]
***Research, Netherlands,*** ***doctors, trainees, consultants, 2007***

The aspirations of the National Service Framework and the NHS Plan will come to nothing if there are not sufficient trained and experienced staff to implement them, and senior staff are increasingly looking forward to early retirement rather than the continued satisfactions of working within the NHS [60]
***Commentary, UK, psychiatrists, medical students, trainees, consultants, 2002***

Why did Julien die and what could have been done to save him? His father has used his own medical training and experience and spent thousands of hours searching for answers. […] and describes the way clinician addiction is handled in the NHS as “amateurish”. [61]
***Commentary, UK, trainees, 2018***

Line management training [..] focuses on educating managers to be more aware of psychologically healthy and supportive work environments. [..] coaching them to spot signs of poor mental health and learning tips on how their own behaviours towards staff can positively or negatively influence the psychological wellbeing of their staff[62].
***Policy, UK, NHS workforce, 2016***

Some leaders and managers may need to be trained (some more than others) in developing the skills and knowledge to balance the demands of well-being and productivity. Otherwise, there is a significant risk of just adding more KPIs without supporting managers to implement them [63]
***Policy, UK, NHS workforce, 2010***

## **CMOc18:Engagement**

If doctors (and students) are involved in the development and implementation of interventions (C), the recipients are more likely to trust (M1) and feel ownership (M2) of the intervention. As a result, it is more likely to be used (O1) and to be effective (O2).

## **Relevant extracts from papers included in the review**

Trying to steer the NHS from the top is like trying to turn a super-tanker. We would like to see more attention being paid to supporting bottom-up initiatives that resonate with staff and which appeal to their intrinsic motivation to care for patients.[64]
***Policy, UK, NHS staff, 2017***

Doctors and managers should work together locally to formulate strategies to give trainees greater autonomy over their working patterns, tasks, and environments. We have a duty to empower emerging medical professionals so that they can continue to shape the future of one of our proudest institutions—the NHS.[65]
***Commentary, UK, doctors, trainees, 2018***

the focus should lie on giving students more scope for the individual organization of their studies [30]. Especially, as the university environment and the reduction of study pressures have been proven to contribute to the reduction of health problems[66]
***Research, Germany, medical students, 2016***

collaborative action planning between physicians and organizational leaders is a constructive way to identify solutions and engage physicians in the process.71,72 This approach gives physicians the opportunity to provide input and share their ideas regarding decisions affecting their work and helps physicians and practice leaders work in partnership toward a shared goal[67]
***Commentary, USA, oncology. Consultants, 2016***

## **CMOc19: Evaluation**

If the outcomes of interventions and the wellbeing of the workforce are regularly reviewed and monitored (C1), and commitment to act upon the outcome of these regular review exercises is shown (C2, and CMOc 16), then doctors may feel more supported (M) and engage with efforts to tailor these interventions (O1). This may also lead to greater awareness about vulnerability and wellbeing in the workplace (O2).

## **Relevant extracts from papers included in the review**

a regular iterative process of inquiry and feedback from physicians can identify issues that negatively affect well-being and barriers to improvement. Third, assessment of well-being using reliable and valid instruments further establishes the value and creates a common language that can help physicians and the organization address well-being issues.[68]
***Intervention, USA, physicians, trainees, consultants, 2007***

Assessing levels of stress among residents may […] contribute to a form of intervention. Moreover, the very use of well-being measures among residents could increase their self-awareness and activate helpful and cathartic discussions on stress, coping strategies and vulnerabilities, consequently partly preventing stress symptoms (Levey, 2001)[69]
***Research, France, internal medicine, trainees, consultant, 2017***

Knight et al9 have been the only group to conduct formal surveys of PHP participants. They found correlations between satisfaction and successful recovery that echoed classic ﬁndings in the addictions literature, and underscored the importance of surveying participants as part of the effort to assess treatment engagement and successful completion [33]

***Research, USA, doctors, trainees, consultants 2016***

Collectively, these findings suggest that periodic assessment and feedback may have relatively universal benefit for physicians because it seems to provide useful information both to those who are doing well (affirmation and reassurance) and to encourage behavioral change to those who are struggling.[70]
***Intervention, USA, surgeons, 2012***

1. Boorman, S., *The Final Report of the independent NHS Health and Well-being review,(2009). Department of Health.* NHS health and well-being review–the government response, 2009.

2. Clarke, R.T., Pitcher, A., Lambert, T.W., Goldacre, M.J., *UK doctors’ views on the implementation of the European Working Time Directive as applied to medical practice: a qualitative analysis.* BMJ open, 2014. **4**(2): p. e004390.

3. Beckman, H., *The role of medical culture in the journey to resilience.* Academic medicine : journal of the Association of American Medical Colleges, 2015. **90**(6): p. 710-2.

4. Panagioti, M., Panagopoulou, E., Bower, P., et al., *Controlled interventions to reduce burnout in physicians: A systematic review and meta-analysis.* JAMA Internal Medicine, 2017. **177**(2): p. 195-205.

5. Chambers, C., Frampton, C., Barclay, M., *Presenteeism in the New Zealand senior medical workforce-a mixed-methods analysis.* The New Zealand medical journal, 2017. **130**(1449): p. 10-21.

6. McKevitt, C., Morgan, M., *Illness doesn't belong to us.* Journal of the Royal Society of Medicine, 1997. **90**(9): p. 491-495.

7. Squiers, J.J., Lobdell, K.W., Fann, J.I., DiMaio, J.M., *Physician Burnout: Are We Treating the Symptoms Instead of the Disease?* The Annals of thoracic surgery, 2017. **104**(4): p. 1117-1122.

8. Doran, N., Fox, F., Rodham, K., Taylor, G., Harris, M., *Lost to the NHS: a mixed methods study of why GPs leave practice early in England.* The British journal of general practice : the journal of the Royal College of General Practitioners, 2016. **66**(643): p. e128-35.

9. Ramirez, A.J., Graham, J., Richards, M.A., Cull, A., *Mental health of hospital consultants: the effects of stress and satisfaction at work.* Lancet, 1996. **347**: p. 724-728.

10. Riley, R., Spiers, J., Chew-Graham, C.A., Taylor, A.K., Thornton, G.A., Buszewicz, M., *‘Treading water but drowning slowly’: what are GPs’ experiences of living and working with mental illness and distress in England? A qualitative study.* BMJ open, 2018b. **8**(5): p. e018620.

11. Montgomery, A., Panagopoulou, E., Kehoe, I., Valkanos, E., *Connecting organisational culture and quality of care in the hospital: is job burnout the missing link?* Journal of Health Organization and Management, 2011. **25**(1): p. 108-23.

12. Gazelle, G., Liebschutz, J.M., Riess, H., *Physician burnout: coaching a way out.* Journal of general internal medicine, 2015. **30**(4): p. 508-13.

13. Shiralkar, M.T., Harris, T.B., Eddins-Folensbee, F.F., Coverdale, J.H., *A systematic review of stress-management programs for medical students.* Academic psychiatry : the journal of the American Association of Directors of Psychiatric Residency Training and the Association for Academic Psychiatry, 2013. **37**(3): p. 158-64.

14. Hochberg, M.S., Berman, R.S., Kalet, A.L., Zabar, S.R., Gillespie, C., Pachter, H.L., *The stress of residency: recognizing the signs of depression and suicide in you and your fellow residents.* American journal of surgery, 2013. **205**(2): p. 141-6.

15. Carvour, M.L., Ayyar, B.K., Chien, K.S., Ramirez, N.C., Yamamoto, H., *A Patient-Centered Approach to Postgraduate Trainee Health and Wellness: An Applied Review and Health Care Delivery Model.* Academic medicine : journal of the Association of American Medical Colleges, 2016. **91**(9): p. 1205-10.

16. Department of Health, *Mental health and ill health in doctors*. 2008: London, UK.

17. Epstein, R.M., Krasner, M.S., *Physician resilience: what it means, why it matters, and how to promote it.* Academic medicine : journal of the Association of American Medical Colleges, 2013. **88**(3): p. 301-3.

18. McKenna, K.M., Hashimoto, D.A., Maguire, M.S., Bynum, W.E.t., *The Missing Link: Connection Is the Key to Resilience in Medical Education.* Academic medicine : journal of the Association of American Medical Colleges, 2016. **91**(9): p. 1197-9.

19. Davies, S.R., Meerton, M., Rost, F., Garelick, A.I., *A sea change for sick doctors - how do doctors fare after presenting to a specialist psychotherapy service?* Journal of Mental Health, 2016. **25**(3): p. 238-244.

20. Bar-Sela, G., Lulav-Grinwald, D., Mitnik, I., *"Balint group" meetings for oncology residents as a tool to improve therapeutic communication skills and reduce burnout level.* Journal of cancer education : the official journal of the American Association for Cancer Education, 2012. **27**(4): p. 786-9.

21. Gunasingam, N., Burns, K., Edwards, J., Dinh, M., Walton, M., *Reducing stress and burnout in junior doctors: the impact of debriefing sessions.* Postgraduate medical journal, 2015. **91**(1074): p. 182-7.

22. McKinley, T.F., Boland, K.A., Mahan, J.D., *Burnout and interventions in pediatric residency: A literature review.* Burnout Research, 2017. **6**: p. 9-17.

23. Mehta, D.H., Perez, G.K., Traeger, L., et al., *Building Resiliency in a Palliative Care Team: A Pilot Study.* Journal of pain and symptom management, 2016. **51**(3): p. 604-8.

24. Foster, E., Biery, N., Dostal, J., Larson, D., *RAFT (Resident Assessment Facilitation Team): supporting resident well-being through an integrated advising and assessment process.* Family medicine, 2012. **44**(10): p. 731-4.

25. Feld, J., Heyse-Moore, L., *An evaluation of a support group for junior doctors working in palliative medicine.* The American journal of hospice & palliative care, 2006. **23**(4): p. 287-96.

26. Benson, J., Magraith, K., *Compassion fatigue and burnout: the role of Balint groups.* Australian family physician, 2005. **34**(6): p. 497-8.

27. Haward, R., Amir, Z., Borrill, C., et al., *Breast cancer teams: The impact of constitution, new cancer workload, and methods of operation on their effectiveness.* British Journal of Cancer, 2003. **89**(1): p. 15-22.

28. Tucker, T., Bouvette, M., Daly, S., Grassau, P., *Finding the sweet spot: Developing, implementing and evaluating a burn out and compassion fatigue intervention for third year medical trainees.* Evaluation & Program Planning, 2017. **65**: p. 106-112.

29. West, C.P., Dyrbye, L.N., Rabatin, J.T., et al., *Intervention to promote physician well-being, job satisfaction, and professionalism: a randomized clinical trial.* JAMA internal medicine, 2014. **174**(4): p. 527-33.

30. Lefebvre, D.C., *Perspective: Resident physician wellness: a new hope.* Academic medicine : journal of the Association of American Medical Colleges, 2012. **87**(5): p. 598-602.

31. Bakker, A.B., Schaufeli, W.B., Sixma, H.J., Bosveld, W., van Dierendonck, D., *Patient demands, lack of reciprocity, and burnout: a five-year longitudinal study among general practitioners.* Journal of Organizational Behavior, 2000. **21**(4): p. 425-441.

32. Shapiro, J., Galowitz, P., *Peer Support for Clinicians: A Programmatic Approach.* Academic medicine : journal of the Association of American Medical Colleges, 2016. **91**(9): p. 1200-4.

33. Sanchez, L.T., Candilis, P.J., Arnstein, F., et al., *Effectiveness of a Unique Support Group for Physicians in a Physician Health Program.* Journal of psychiatric practice, 2016. **22**(1): p. 56-63.

34. Hamader, G., Noehammer, E., *Prevention of anxiety, depression and burnout during medical studies and residency training (experts' opinion, medical students' and young doctors' point of view).* Noehammer, Elisabeth [Ed] (2013) Psychology of well-being: Theory, perspectives and practice (pp 33-42) xii, 229 pp Hauppauge, NY, US: Nova Science Publishers; US, 2013: p. 33-42.

35. George, D.R., Dellasega, C., Whitehead, M., *Facebook stress management group for Year 1 medical students.* Medical Education, 2012. **46**(11): p. 1118.

36. McNeill, K.G., Kerr, A., Mavor, K.I., *Identity and norms: the role of group membership in medical student wellbeing.* Perspectives on Medical Education, 2014. **3**(2): p. 101-12.

37. Oczkowski, S., *Virtuous laughter: we should teach medical learners the art of humor.* Critical care (London, England), 2015. **19**: p. 222.

38. Schapira, L., Meisel, J.L., Srivastava, R., *For Our Patients, for Ourselves: The Value of Personal Reflection in Oncology.* American Society of Clinical Oncology Educational Book, 2017. **37**: p. 765-770.

39. Haizlip, J., May, N., Schorling, J., Williams, A., Plews-Ogan, M., *Perspective: the negativity bias, medical education, and the culture of academic medicine: why culture change is hard.* Academic medicine : journal of the Association of American Medical Colleges, 2012. **87**(9): p. 1205-9.

40. Flowers, L.K., *The missing curriculum: experience with emotional competence education and training for premedical and medical students.* Journal of the National Medical Association, 2005. **97**(9): p. 1280-7.

41. Slavin, S.J., Hatchett, L., Chibnall, J.T., Schindler, D., Fendell, G., *Helping medical students and residents flourish: A path to transform medical education.* Academic Medicine, 2011. **86**(11): p. e15.

42. Gerada, C., *For doctors with mental illness, ‘help me’ can be the hardest words*. 2018, <https://www.theguardian.com/commentisfree/2018/jun/06/doctors-mental-health-problems-taboo> (last accessed 20/08/2019): The Guardian

43. Barbosa, P., Raymond, G., Zlotnick, C., Wilk, J., Toomey, R., 3rd, Mitchell, J., 3rd, *Mindfulness-based stress reduction training is associated with greater empathy and reduced anxiety for graduate healthcare students.* Education for health (Abingdon, England), 2013. **26**(1): p. 9-14.

44. Davies, S.R., Meerton, M., Rost, F., Garelick, A.I., *A sea change for sick doctors - how do doctors fare after presenting to a specialist psychotherapy service?* Journal of mental health (Abingdon, England), 2016. **25**(3): p. 238-44.

45. Meerten, M., Rost, F., Bland, J., Garelick, A.I., *Self-referrals to a doctors' mental health service over 10 years.* Occupational medicine (Oxford, England), 2014. **64**(3): p. 172-6.

46. Wolf, T., *Stress, coping and health: Enhancing well-being during medical school.* Medical Education, 1994. **28**(1): p. 8-17.

47. Carter, Y., Peile, E., *Selecting and supporting contented doctors.* British Medical Journal, 2005. **330**(7486): p. 269-270.

48. Ripp, J.A., Privitera, M.R., West, C.P., et al., *Well-Being in Graduate Medical Education: A Call for Action.* Academic medicine : journal of the Association of American Medical Colleges, 2017. **92**(7): p. 914-917.

49. Horsfall, S., *Doctors who commit suicide while under GMC fitness to practice investigation.* General Medical Council, 2014.

50. Schmitz, G.R., Clark, M., Heron, S., et al., *Strategies for coping with stress in emergency medicine: Early education is vital.* Journal of Emergencies Trauma & Shock, 2012. **5**(1): p. 64-9.

51. Ey, S., Moffit, M., Kinzie, J.M., Brunett, P.H., *Feasibility of a Comprehensive Wellness and Suicide Prevention Program: A Decade of Caring for Physicians in Training and Practice.* Journal of graduate medical education, 2016. **8**(5): p. 747-753.

52. Firth-Cozens, J., *Improving the health of psychiatrists.* Advances in Psychiatric Treatment, 2007. **13**(3): p. 161-168.

53. Blais, R., Safianyk, C., Magnan, A., Lapierre, A., *Physician, heal thyself: Survey of users of the Quebec Physicians Health Program.* Canadian family physician Medecin de famille canadien, 2010. **56**(10): p. e383-9.

54. Chaukos, D., Chad-Friedman, E., Mehta, D.H., et al., *Smart-r: A prospective cohort study of a resilience curriculum for residents by residents.* Academic Psychiatry, 2017: p. No Pagination Specified.

55. Ey, S., Moffit, M., Kinzie, J.M., Choi, D., Girard, D.E., *"If you build it, they will come": attitudes of medical residents and fellows about seeking services in a resident wellness program.* Journal of Graduate Medical Education, 2013. **5**(3): p. 486-92.

56. Moutier, C., Norcross, W., Jong, P., et al., *The suicide prevention and depression awareness program at the University of California, San Diego School of Medicine.* Academic medicine : journal of the Association of American Medical Colleges, 2012. **87**(3): p. 320-6.

57. Department of Health, *NHS Health and Wellbeing Review: Interim Report*. 2009, The Stationary Office London.

58. Schneider, S., Kingsolver, K., Rosdahl, J., *Physician coaching to enhance well-being: a qualitative analysis of a pilot intervention.* Explore (New York, N.Y.), 2014. **10**(6): p. 372-9.

59. Prins, J.T., Hoekstra-Weebers, J.E.H.M., Gazendam-Donofrio, S.M., et al., *The role of social support in burnout among Dutch medical residents.* Psychology, Health & Medicine, 2007. **12**(1): p. 1-6.

60. Roberts, G., Moore, B., Coles, C., *Mentoring for newly appointed consultant psychiatrists.* Psychiatric Bulletin, 2002. **26**(3): p. 106-109.

61. Dyer, C., *Julien Warshafsky: how this doctor died and what it tells us about the system that failed him.* BMJ, 2018. **361**: p. k2564.

62. NHS England, *NHS Staff Health and Wellbeing: CQUIN Supplementary Guidance; 2016*. 2016.

63. Robertson, I., Cooper, C., *The Boorman Report on the Health and Well-Being of NHS Staff: Practical advice for implementing its recommendations.* 2010.

64. Cornwell, J., Fitzsimons, B., *Behind Closed Doors. Can we expect NHS staff to be the shock absorbers of a system under pressure?* 2017, The Point of Care Foundation.

65. Penfold, R., *Why junior doctors need more autonomy.* BMJ, 2018. **363**.

66. Bugaj, T., Cranz, A., Junne, F., Erschens, R., Herzog, W., Nikendei, C., *Psychosocial burden in medical students and specific prevention strategies.* Mental Health and Prevention, 2016. **4**(1): p. 24-30.

67. Hlubocky, F.J., Back, A.L., Shanafelt, T.D., *Addressing Burnout in Oncology: Why Cancer Care Clinicians Are At Risk, What Individuals Can Do, and How Organizations Can Respond.* American Society of Clinical Oncology educational book. American Society of Clinical Oncology. Meeting, 2016. **35**: p. 271-9.

68. Dunn, P.M., Arnetz, B.B., Christensen, J.F., Homer, L., *Meeting the imperative to improve physician well-being: assessment of an innovative program.* Journal of general internal medicine, 2007. **22**(11): p. 1544-52.

69. Myszkowski, N., Villoing, B., Zenasni, F., Jaury, P., Boujut, E., *Monitoring stress among internal medicine residents: an experience-driven, practical and short measure.* Psychology, Health & Medicine, 2017. **22**(6): p. 719-726.

70. Shanafelt, T.D., Kaups, K.L., Nelson, H., et al., *An interactive individualized intervention to promote behavioral change to increase personal well-being in US surgeons.* Annals of surgery, 2014. **259**(1): p. 82-8.
